# Short-term heat acclimation preserves knee extensor torque but does not improve 20 km self-paced cycling performance in the heat

**DOI:** 10.1007/s00421-021-04744-y

**Published:** 2021-06-19

**Authors:** John O. Osborne, Ian B. Stewart, David N. Borg, Kenneth W. Beagley, Robert L. Buhmann, Geoffrey M. Minett

**Affiliations:** 1 School of Sport Sciences, UiT The Arctic University of Norway, Tromsø, Norway; 2 School of Exercise and Nutrition Sciences, Queensland University of Technology (QUT), Brisbane, Australia; 3 Institute of Health and Biomedical Innovation, Queensland University of Technology (QUT), Brisbane, Australia; 4 School of Biomedical Sciences, Queensland University of Technology (QUT), Brisbane, Australia; 5 The Hopkins Centre, Menzies Health Institute Queensland, Griffith University, Brisbane, Australia; 6 School of Health and Sport Sciences, University of the Sunshine Coast, Maroochydore, Australia

**Keywords:** Athletic performance, Muscle fatigue, Thermotolerance, Heat stress, Hyperthermia

## Abstract

**Purpose:**

This study investigated the effect of 5 days of heat acclimation training on neuromuscular function, intestinal damage, and 20 km cycling (20TT) performance in the heat.

**Methods:**

Eight recreationally trained males completed two 5-day training blocks (cycling 60 min day^−1^ at 50% peak power output) in a counter-balanced, cross-over design, with a 20TT completed before and after each block. Training was conducted in hot (HA: 34.9 ± 0.7 °C, 53 ± 4% relative humidity) or temperate (CON: 22.2 ± 2.6 °C, 65 ± 8% relative humidity) environment. All 20TTs were completed in the heat (35.1 ± 0.5 °C, 51 ± 4% relative humidity). Neuromuscular assessment of knee extensors (5 × 5 s maximum voluntary contraction; MVC) was completed before and after each 20TT and on the first and last days of each training block.

**Results:**

MVC torque was statistically higher after 5 days of HA training compared to CON (mean difference = 14 N m [95% confidence interval; 6, 23]; *p* < 0.001; *d* = 0.77). However, 20TT performance after 5 days of HA training was not statistically different to CON, with a between-conditions mean difference in the completion time of 68 s [95% confidence interval; − 9, 145] (*p* = 0.076; *d* = 0.35).

**Conclusion:**

Short-term heat acclimation training may increase knee extensor strength without changes in central fatigue or intestinal damage. Nevertheless, it is insufficient to improve 20 km self-paced cycling performance in the heat compared to workload-matched training in a temperate environment. These data suggest that recreationally trained athletes gain no worthwhile performance advantage from short-term heat-training before competing in the heat.

**Supplementary Information:**

The online version contains supplementary material available at 10.1007/s00421-021-04744-y.

## Introduction

Exercise performance in hot conditions is compromised by impaired metabolic heat loss that raises thermal strain and evokes fatigue (Nybo et al. 2014). Repeated exposure to heat stress and the resultant prolonged elevation of core temperature facilitates a host of beneficial physiological adaptations. This heat acclimation (HA) includes enhanced sweat rates and lower exercising core temperatures that improve subsequent aerobic performance in the heat (Sawka et al. 2011). In comparison to traditional HA protocols, short-term approaches (e.g., ≤ 7 days) are proposed as a practical, time-efficient alternative for athletes when tapering before competition (Racinais and Périard 2020), while still partially inducing beneficial physiological adaptations (Garrett et al. 2011; Chalmers et al. 2014; Daanen et al. 2018). These adaptions are also present in moderately trained and recreational athletes who undertake HA training (Garrett et al. 2009, 2011, 2019; Ashley et al. 2015).

When athletes experience high core temperatures, skeletal muscle excitability may be compromised due to central and peripheral mechanisms (Nybo and Nielsen 2001; Cheung 2007). However, conflicting evidence for a causal effect suggests that additional mechanistic factors may also be involved (Nybo and González-Alonso 2015). One possible mechanism proposes that decreased splanchnic blood flow during exercise-heat stress and the resultant damage to the intestinal barrier increases inflammatory cytokine expression (Lambert 2004; Osborne et al. 2019b). While the exact role that inflammatory cytokines might play in the modulation of central fatigue is poorly understood, cytokine-mediated alterations in neurotransmitter levels could potentially influence descending drive (Davis and Bailey 1997; Vargas and Marino 2014). Therefore, HA-induced changes in cytokine expression may modify excitatory input to skeletal muscle and possibly contribute to post-acclimation exercise performance improvements, however, this concept has yet to be investigated.

The observed improvements in exercise performance following short-term HA are thought to primarily stem from rapid cardiovascular adaptions, such as plasma volume expansion and redistribution of blood volume, which relieves cardiovascular strain during exercise-heat stress (Nybo et al. 2014; Périard et al. 2015). Superior cardiac output is also thought to maintain gut blood flow and preserves intestinal barrier integrity (Lambert 2004) as well as reducing thermal stress to intestinal epithelial cells (Moseley et al. 1994). HA training has also been found to upregulate heat shock protein 70 expression (Sawka et al. 2011; Nava and Zuhl 2020), which appears to strengthen intestinal tight junctions (Moseley et al. 1994) and attenuate intestinal barrier permeability (Lambert 2004; Kuennen et al. 2011). Therefore, it is reasonable to posit that HA training, through one or both of these mechanisms, may improve exercise performance via maintenance of gut integrity and limiting central fatigue during heat stress (Osborne et al. 2019b).

Despite short-term HA studies (Garrett et al. 2011; Chalmers et al. 2014; Ashley et al. 2015; Daanen et al. 2018) demonstrating physiological, perceptual and performance improvements, the effect of such HA training on neuromuscular function remains equivocal. For example, voluntary activation and strength is unaffected following repeated passive (non-exercise) HA trials, while the skeletal contractile function is either improved (Racinais et al. 2017a) or unchanged (Brazaitis and Skurvydas 2010). To our knowledge, the only active (exercise-induced) HA study that has explored this topic did not measure neuromuscular function post-intervention or use a control group, confounding the evaluation of their findings (Wingfield et al. 2016). Furthermore, as changes in neuromuscular function (i.e., descending drive) seemingly modulate exercise performance (Taylor et al. 2016), it could be argued that HA-induced adaptations in neuromuscular function might influence self-paced performance in the heat. However, there has been limited research on this topic and the effect of several consecutive days of exercise-heat stress on neuromuscular function and self-paced exercise performance is unknown.

This study examined the effects of 5 consecutive days of HA training on self-paced performance in the heat and neuromuscular function in recreationally trained athletes. It was hypothesised that HA training would: (1) improve self-paced exercise performance in the heat; (2) reduce intestinal damage and inflammation; and (3) attenuate the development of central fatigue and preserve neuromuscular function.

## Methods

### Participants

Eight recreationally trained males completed this study (age 26.5 ± 1.8 years; height 181 ± 9 cm; nude mass 82 ± 12 kg; peak oxygen consumption [$\dot{V}{\text{O}}_{{2{\text{peak}}}}$] 49.3 ± 4.9 mL kg^−1^ min^−1^; peak power output [PPO]: 347 ± 56 W; peak heart rate [HR] 187 ± 13 beats min^−1^). Participants’ usual weekly training activities were 3.6 ± 1.3 training sessions and 191 ± 63 training minutes per week and were classified as recreationally trained or trained (performance level 2 or 3; De Pauw et al. 2013). Participants were non-smokers, free of injury or illnesses with no history of gastrointestinal or kidney diseases. Queensland University of Technology Human Research Ethics Committee approved the study (Approval: 1700000651), and all participants provided written informed consent.

### Experimental overview

The study followed a counter-balanced, cross-over design, with participants completing two, 5-day cycle training blocks (60 min day^−1^) in a hot (HA) and control (CON) environment. The HA condition was regulated in a custom-built environmental climate chamber (dimensions: 4 m length; 3 m width; 2.5 m height; wind speed = 4.7 km h^−1^) at 34.9 ± 0.7 °C and 53 ± 4% relative humidity (RH). The CON environment was a climate-controlled laboratory at 22.2 ± 2.6 °C and 65 ± 8% RH. Participants were familiarised with all testing procedures and completed a practice 20 km time trial (20TT). Participants undertook a 20TT in the heat (35.1 ± 0.5 °C, 51 ± 4% RH) 2 days before and after each training block, with the day immediately before and after the 5-day training period considered a rest day. Participants were asked to maintain hydration during the 5-day training periods and arrive for all 20TT and training sessions adequately hydrated. Participants abstained from caffeine and alcohol for 12 h, and strenuous exercise for 24 h before each 20TT. Fluid consumption and external fan cooling were prohibited during all cycling sessions. There were at least 34 days between the two conditions (mean ± SD = 59 ± 24 days). Training sessions and time trials were matched for the time of day within participants (± 2 h).

A self-paced performance task (20TT) was selected for greater external validity (Currell and Jeukendrup 2008; Marino 2010), in comparison to fixed-intensity or time-to-exhaustion tests which are often used in HA research (Tyler et al. 2016; Benjamin et al. 2019). The use of a 20TT performance task allowed evaluation of the HA training effect on self-regulated work in the heat (Tucker and Noakes 2009), an area which has not been as extensively studied (Tyler et al. 2016; Benjamin et al. 2019).

The HA training protocol—5 days of cycling at 50% PPO, for 60 min in 35 °C and 50% RH—was selected following examination of the published short-term HA literature (Sunderland et al. 2008; Brade et al. 2013; Chen et al. 2013; Chalmers et al. 2014). Particular attention was paid to the recreational fitness level of the cohort in the current study, as well as the implementation of fluid prohibition during training to induce additional strain and adaptations (Garrett et al. 2011). The necessary (minimum) wash-out time between conditions was determined from previous HA adaptation and decay literature (Garrett et al. 2009; Daanen et al. 2018).

### $\dot{V}{\text{O}}_{{2{\text{peak}}}}$ and peak power output protocol

Five days before each training block, an incremental cycling test (Excalibur Sport; Lode, Groningen, Netherlands) was conducted in temperate conditions for the measurement of $\dot{V}{\text{O}}_{{2{\text{peak}}}}$ and peak power output (PPO). Participants began each test at 75 W and the load increased in 25 W min^−1^ steps until volitional fatigue. A calibrated gas analyser (TrueOne 2400; ParvoMedics, Salt Lake City, USA) collected breath-by-breath measurements, with data averaged over a 15 s epoch and the peak values considered as the $\dot{V}{\text{O}}_{{2{\text{peak}}}}$. PPO was calculated as previously described by De Pauw et al. (2013).

### 20TT protocol

Before the 20TT, a mid-stream urine sample and venous blood samples were collected, and pre-exercise neuromuscular assessments completed. Arrival hydration status was assessed through urine specific gravity (PAL-10S; Atago Co. Ltd, Tokyo, Japan) and osmolality (Osmomat 030; Gonotec, Berlin, Germany). Nude mass was recorded (WB-110AZ; Tanita Corp., Tokyo, Japan), and a rectal thermistor (449H; Henleys Medical, Welwyn Garden City, UK) inserted 12 cm beyond the anal sphincter for measurements of rectal temperature (*T*_re_; T-Tec 7 3E-RF; Temperature Technology, Adelaide, Australia). A HR monitor was fitted (Polar Team^2^; Polar Electro Oy, Kempele, Finland) and iButton thermocrons (DS1922L-F50 iButtons; Maxim Integrated, San Jose, USA) were taped (Leuko Sportstape Premium; Beiersdorf, Hamburg, Germany) to four sites: the posterior neck (28% weighting), inferior border of the right scapula (28%), posterior left hand (16%), and mid-anterior right shin (28%) for the retrospective calculation of mean skin temperature (*T*_sk_) (ISO 9886,2004). Participants rested supine for 20 min, in a quiet, dimly lit room (21.2 ± 0.9 °C, 60 ± 4% RH) to record baseline *T*_re_, *T*_sk_ and HR; considered the average between 10 and 20 min.

After baseline recording, participants donned their cycling attire and entered the chamber to commence a flat course 20TT (Velotron Pro; RacerMate Inc., Seattle, USA). Cycling started from a stationary, seated position. Apart from an elapsed distance and the selected resistance in gear-inches, all feedback was withheld. Participants were instructed to complete the distance as fast as possible. No encouragement was provided. During cycling, HR, *T*_re_ and *T*_sk_ were continuously recorded. A rating of perceived exertion (RPE) was collected every 2 km using Borg’s 6–20 scale (Borg 1998). Neuromuscular testing commenced within 2 min of finishing the 20TT, and a nude mass was recorded for sweat loss calculations.

### Training day protocol

Upon arrival at the laboratory, participants provided a mid-stream urine sample for hydration status assessment, as previously described. Before each training session, nude mass was recorded, and participants were instrumented with a rectal thermistor, HR monitor and four iButtons, as described above. Participants then cycled at 50% PPO (Wattbike Pro; Wattbike Ltd, Nottingham, United Kingdom) for 60 min in their allocated environment. Each participants’ cycling attire, pedals and ergometer settings remained consistent across all training sessions. The HR, *T*_re_ and *T*_sk_ were continuously recorded during training, and the RPE recorded every 10 min. Participants exited the chamber within 1 min of exercise completion and towelled-dried before post-exercise nude mass was recorded. Fluid consumption was prohibited during the training sessions to stress fluid homeostasis and facilitate enhanced acclimation, as recommended by Garret et al. (2011).

### Neuromuscular function

The neuromuscular function of the right quadriceps muscles (i.e., vastus lateralis and vastus medialis) was assessed before and after every 20TT, and on day 1 and day 5 of training. Procedures for neuromuscular testing followed standard laboratory methods (Osborne et al. 2019a). Participants performed a standardised warm-up of eight isometric knee extensions (90° knee flexion) of increasing intensity, rested for 3 min, and then completed a set of five, 5 s maximal voluntary isometric knee extensions (MVCs), with a 30 s rest between each repetition. Self-adhesive gel electrodes (Pals; Axelgaard Manufacturing Co. Ltd., Fallbrook, CA) were placed over the right femoral triangle (cathode) and gluteal fold (anode). Stimuli were applied via a Digitimer DS7AH stimulator (Digitimer Ltd., Welwyn Garden City, Hertfordshire, England) using a single, 100 µs pulse. A twitch ramp procedure was used to determine the required current for a twitch torque plateau and was increased by an additional 10% during MVCs to ensure supramaximal stimulation (Osborne et al. 2019a). During MVCs, electrical stimuli were manually triggered upon voluntary torque plateau, and participants continued to contract following the delivery of the stimulus. A second stimulus was triggered within 2–3 s of MVC cessation. Contractions were rejected when: torque was < 95% of the maximum; a stimulus was not delivered during a plateau; or if post-stimulation voluntary torque exceeded pre-stimulation values. In our laboratory, familiarised participants demonstrate substantially reliable performance in this task, with an intraclass correlation (2,1) of 0.94 and 0.94 for voluntary activation and MVC torque, respectively (Osborne et al. 2019a; b).

Evoked twitch contractile properties and surface electromyography (EMG) data were collected using previously published methods (Osborne et al. 2019a). All sites were shaved, abraded and swabbed with alcohol. Electrodes (10 mm diameter Ambu Blue Sensor N-00-S; Ambu A/S, Ballerup, Denmark) were placed in parallel (20 mm inter-electrode distance) with the presumed direction of the muscle fibres over the most prominent portion of the muscle belly along the line connecting the anterior superior iliac spine and medial patella for the vastus medialis and lateral patella for the vastus lateralis. MVC torque and peak EMG amplitude were considered the 100 ms average around the peak value before electrical stimulation. Voluntary activation was calculated using the following equation (Allen et al. 1995):

$${\text{Voluntary activation}}\;\% = \left[ {1 - \left( {{\text{superimposed twitch}}/{\text{potentiated twitch}}} \right)} \right] \times 100$$

The superimposed twitch is the difference between MVC torque and the maximum torque evoked by the electrical stimulus during the MVC. The potentiated twitch is the peak torque response elicited in resting muscle 2–3 s following the MVC. Maximal M-waves and V-waves were collected from the vastus medialis and vastus lateralis during MVCs where stimulation was applied. The peak-to-peak amplitude of M-waves and V-waves were automatically determined in LabChart (v8.1.5; AD Instruments, Bella Vista, Australia). EMG amplitudes and V-waves were normalised to maximal M-waves to control for changes in membrane excitability due to fatigue (Girard et al. 2018) and expressed as a percentage. The three repetitions exhibiting the greatest torque value for each assessment were used for analysis.

### Blood measures

Venous blood samples were collected before and after the 20TT from an antecubital venepuncture on seated participants. Whole blood was used to determine haemoglobin (HemoCue Model 201 + , Angelholm, Sweden) and haematocrit (12,000 RPM, 10 min, 24 °C). Haematocrit was measured in triplicate (CV = 1.8%) and haemoglobin in duplicate (CV = 0.9%). Mean haematocrit and haemoglobin values were used to calculate plasma volume (Dill and Costill 1974). An SST tube was collected for serum osmolality analysis (Osmomat 030; Gonotec, Berlin, Germany). EDTA vacutainer tubes (BD Pty. Ltd., Macquarie Park, Australia) were centrifuged (3500 RPM, 15 min, 4 °C), aliquoted and frozen (− 80 °C) for analysis of inflammatory markers.

Blood concentrations of TNF-α (EK-0001; elisakit.com, Melbourne, Australia), IL-6 (HS600B; R&D Systems, Minneapolis, USA) and I-FABP (EHFABP2; Thermo Scientific, Fredrick, USA) were determined using quantitative sandwich enzyme-linked immunoassays (ELISA). Intra-assay variance was calculated for TNF-α (CV = 8.8%), IL-6 (CV = 8.0%) and I-FABP (CV = 6.6%). Inter-assay variance for TNF-α was CV = 8.8% and for I-FABP was CV = 7.4%. All samples were diluted to reduce interference (TNF-α, 1:3; IL-6, 1:4; I-FABP, 1:2), and absorbance was read using a SpectroStar Nano (BMG Labtech, Offenburg, Germany) with wavelength corrections for plate imperfections. Due to equipment limitations, IL-6 concentrations were only analysed at post-cycling timepoints. Blood markers were selected based on previous literature, as strenuous exercise in the heat has been linked to gastrointestinal damage (I-FABP) (Lambert 2008) and the resultant release of pro-inflammatory cytokines (TNF-α and IL-6) (Lim and Mackinnon 2006), which may modulate the development of central fatigue (Vargas and Marino 2014).

### Data analysis

All analyses were performed in R v4.0.2 (R Core Team 2020). The R packages used are listed in Supplement 1. Power was a priori simulated and assumed an initial 20 km completion time of 2169 s, taken from previous reliability data (Borg et al. 2018). The effect of HA performance was set at 3.8% (83 s improvement), calculated as the mean HA improvement (+ 4.5%; + 98 s) in similar HA research (Lee et al. 2016; Willmott et al. 2016; Wingfield et al. 2016), minus the average performance improvement of the control group (0.7%, 15 s) (Lee et al. 2016; Willmott et al. 2016). Standard deviation of the random intercept (participant) was set at 120 s. For eight participants and α level of 5%, power was 83.2%. See code in Supplement 1 for further detail.

An equivalence test procedure was performed using the *TOSTER* package (Lakens 2017) to determine if the primary outcome variable, the between-groups difference in 20TT completion time, lay within set equivalence bounds of a worthwhile performance change (0 ± 95 s), based on previously published data (Borg et al. 2018).

Data were analysed using hierarchical regression. Baseline/pre-cycling 20TT variable models included *condition* (HA or CON), *test* (pre- or post-5-day training intervention) and interactions as fixed effects; and pre–post-cycling 20TT variable models included *condition*, *test*, *time* (before or after 20TT) and interactions as fixed effects. Hierarchical regression models were fit to physiological variables during cycling, with the selected model having the smallest Bayesian Information Criteria (BIC) value. Candidate models considered non-linear terms for *distance* (during a 20TT) or *cycletime* (during the 60 min training), *condition*, *test* or *day* (training day 1 or 5), and their interactions as fixed effects. The difference in power output and cadence between conditions was modelled, with the *test*, *distance,* and their interaction included as fixed effects. All baseline and pre–post-cycling models included a random intercept or intercept and slope (*condition*) for each participant, and cycling models a random intercept or intercept and slope (*distance* or *cycletime*) for each participant. RPE was modelled using beta regression to account for the skewed distribution of the errors.

Post hoc tests were made at 5 min intervals during training and every 2 km during 20TT, with *p*-values Bonferroni corrected. Cohen’s *d* was calculated, using the pooled standard deviation as the denominator, and interpreted as small 0.20, medium 0.50 and large ≥ 0.80 (Cohen 1992). Cliff’s delta (*δ*_Cliff_) (Cliff 1996), a non-parametric standardised effect size measure (range: − 1 to 1), was used for RPE. *δ*_Cliff_ was interpreted as small 0.147, medium 0.33, and large 0.474 (Romano et al. 2006). Standardised effect sizes (and 95% CI) were calculated using the *effsize* package. For all tests, α was set at 5%. Data are reported as the mean, or mean difference (MD), and 95% confidence interval (CI), unless otherwise stated. Figures were generated using the *ggplot2* package (Wickham 2016).

## Results

### 20TT pre- and post-intervention

#### Cycling performance

**Fig. 1:**
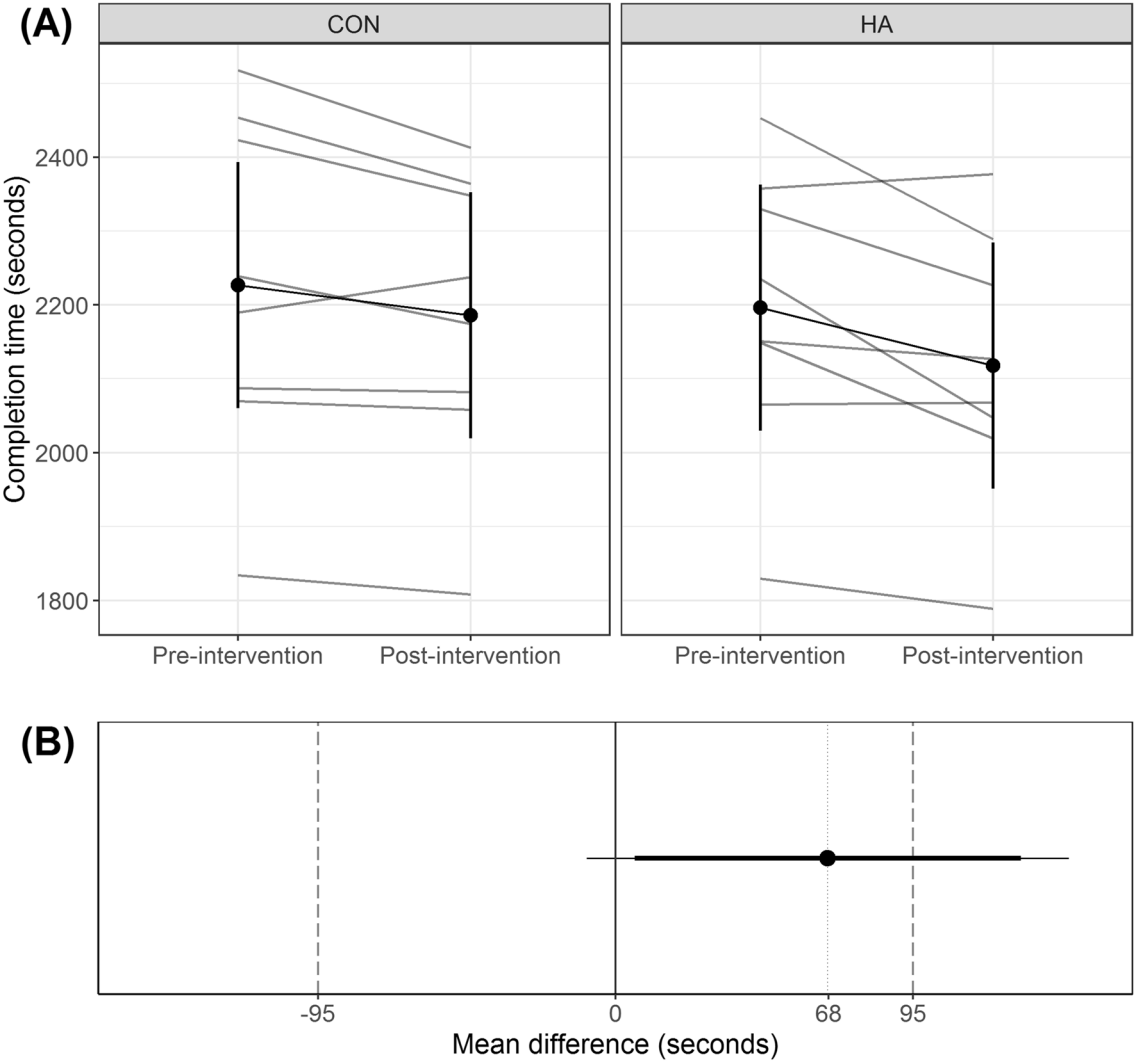
**A** Mean and 95% confidence interval (vertical black lines) 20 km time trial completion time before and after a 5-day training intervention in a hot (HA) or temperate (CON) environment, where the grey lines indicate individual participants. **B** Mean difference in completion time between HA and CON in the post-intervention time trial, with 90% (thick black line) and 95% (thin black line) confidence intervals, where the grey dashed vertical lines indicate the equivalence bounds for a worthwhile change in performance (i.e., 0 ± 95 s)

Post-intervention 20TT completion time for HA was 68 s [− 9, 145] faster than CON, though this was not statistically different (*p* = 0.076; Fig. 1A), nor statistically equivalent (*p* = 0.215; Fig. 1B). A small effect was observed for the post-intervention 20TT time, between conditions (*d* = 0.35 [− 0.02, 0.71]). There was no *test* by *condition* effect on cycling performance (*p* = 0.336; Supplement 2—Output 1). The 20TT completion time change (pre-20TT versus post-20TT time) for HA was 37 s [− 44, 118] faster than CON, but not statistically different (*p* = 0.312).

The between-conditions difference in power output and cadence (Supplement 2—Output 1) was not statistically different (*p* = 0*.*26–0.73). The mean between-conditions 20TT power output and cadence are shown in Supplement 3—Figs. 1A & 2, and individual 20TT power outputs for each participant are shown in Supplement 3— Figs. 1B.

#### Neuromuscular measures

Post-intervention HA MVC was statistically higher than post-intervention CON (*p* < 0.001; MD = 14 N m [6, 23]; *d* = 0.77 [0.26, 1.28]; Table 1) and pre-intervention HA (*p* < 0.001; MD = 17 N m [9, 25]; *d* = 0.88 [0.38, 1.38]). Pre-intervention EMG-M_max_ was statistically higher in CON compared to HA (*p* = 0*.*004). Voluntary activation, Pt, CD, RTD, RR, 0.5RT, V-wave and V-M_max_ were not statistically different between conditions at any point (Supplement 2—Output 2; Supplement 4—Table 1).

**Table 1:** Neuromuscular variables before and after time trial performance

Variable	Condition	Pre-intervention		Post-intervention	
		Pre-cycling	Post-cycling	Pre-cycling	Post-cycling
MVC (N m)	CON	255 [219, 291]	219 [183, 255]	240 [204, 276]	226 [190, 262]
	HA	245 [210, 281]	216 [180, 252]	258 [222, 294]^a^	237 [201, 273]^a^
VA (%)	CON	92.6 [88.2, 97.1]	86.8 [82.3, 91.3]	92.8 [88.4, 97.3]	90.5 [86.1, 95.0]
	HA	92.9 [88.5, 97.3]	85.6 [81.2, 90.0]	93.7 [89.2, 98.1]	89.8 [85.4, 94.2]
Pt (N m)	CON	67 [51, 78]	48 [38, 59]	67 [56, 77]	48 [38, 59]
	HA	69 [59, 79]	49 [39, 59]	65 [55, 76]	45 [35, 56]
V-M_max_	CON	0.260 [0.154, 0.365]	0.237 [0130, 0.343]	0.275 [0.170, 0.380]	0.201 [0.093, 0.308]
	HA	0.329 [0.224, 0.434]	0.233 [0.127, 0.340]	0.312 [0.208, 0.417]	0.232 [0.126, 0.339]
EMG-M_max_	CON	61.8 [49.4, 77.4]	52.1 [41.6, 65.1]	58.4 [46.7, 73.0]	52.0 [41.6, 65.0]
	HA	48.9 [39.1, 61.2]^b^	50.3 [40.2, 62.9]	67.2 [53.8, 84.1]	51.1 [40.8, 63.9]

Values reported as mean [95% confidence interval]

*CON* control, *HA* heat acclimation, *MVC* maximum voluntary contraction, *VA* voluntary activation, *Pt* potentiated twitch, *EMG* electromyography

^a^Different to CON within the same time trial (averaged over levels of time)

^b^Different to CON within the same trial, at the same time point

#### Baseline and pre–post-20TT measures

Baseline and pre–post-20TT variables are displayed in Table 2. Baseline *T*_re_, baseline HR, serum osmolality, urine osmolality, urine specific gravity, inflammatory blood markers and pre-cycling body mass were not statistically different between conditions at any point (Table 2; Supplement 2—Output 3). The change in plasma volume after each training block was statistically greater in HA compared to CON (*p* = 0*.*036; MD = 5.0% [0.4, 9.6]; *d* = − 1.52 [− 3.35, 0.32]). Sweat rates in the post-intervention time trial were statistically higher in HA compared to CON (*p* = 0*.*007; MD = 0.40 L h^−1^ [0.13, 0.68]; *d* = 1.82 [0.39, 3.26]). There were no significant between-conditions differences for peak aerobic capacity (absolute or relative $\dot{V}{\text{O}}_{{2{\text{peak}}}}$; *p* = 0.309–0.383) or peak power (*p* = 0.612) before each training block.

**Table 2:** Pre- and post-intervention time trial data

Variable	Time	Pre-intervention		Post-intervention	
		CON	HA	CON	HA
Performance					
Completion time (seconds)		2227 [2060, 2394]	2196 [2030, 2363]	2186 [2019, 2352]	2118 [1951, 2285]
Mean power output (Watts)		197 [152, 242]	203 [158, 248]	205 [160, 250]	221 [175, 266]
Mean cadence (revolutions min^−1^)		92 [85, 100]	93 [86, 101]	92 [84, 99]	92 [84, 99]
Physiological					
Baseline HR (beats min^−1^)	Pre-cycling	55 [50, 60]	53 [47, 58]	50 [45, 55]	50 [44, 55]
Baseline *T*_re_ (°C)	Pre-cycling	37.0 [36.9, 37.2]	37.2 [37.0, 37.3]	37.1 [36.9, 37.2]	37.1 [37.0, 37.3]
Haematological					
PV change (%)	Pre-cycling	–	–	4.0 [1.7, 6.3]	9.0 [5.8, 12.2]
I-FABP (pg mL^−1^)	Pre-cycling	561 [270, 853]	452 [196, 707]	696 [404, 988]	681 [409, 952]
	Post-cycling	934 [663, [1206]	975 [703, 1247]	1373 [1117, 1628]	1418 [1162, 1674]
TNF-α (pg mL^−1^)	Pre-cycling	3.47 [1.91, 5.04]	3.63 [2.13, 5.13]	3.37 [1.73, 5.02]	3.64 [2.14, 5.14]
	Post-cycling	4.88 [3.23, 6.53]	4.44 [2.87, 6.01]	5.62 [4.12, 7.12]	3.59 [2.03, 5.15]
IL-6 (pg mL^−1^)	Post-cycling	2.822 [1.669, 4.772]	4.949 [2.839, 8.627]	1.975 [1.168, 3.339]	3.436 [2.032, 5.811]
Hydration					
Body mass (kg)	Pre-cycling	80.2 [70.5, 91.2]	79.1 [69.6, 90.0]	79.9 [70.3, 90.9]	79.6 [70.0, 90.5]
Sweat rate (L h^−1^)	During cycling	1.29 [0.94, 1.65]	1.20 [0.83, 1.56]	1.27 [0.91, 1.62]	1.67 [1.29, 2.05]^a^
Serum osmolality (mOsm kg^−1^)	Pre-cycling	294 [291, 297]	293 [291, 296]	295 [292, 299]	293 [290, 297]
Urine osmolality (mOsm kg^−1^)	Pre-cycling	777 [384, 1169]	503 [111, 895]	411 [– 46, 868]	425 [34, 817]
Urine specific gravity (AU)	Pre-cycling	1.014 [1.006, 1.022]	1.012 [ 1.004. 1.019]	1.009 [1.002, 1.017]	1.007 [1.000, 1.015]
Peak aerobic capacity					
Relative $\dot{V}{\text{O}}_{{2{\text{peak}}}}$ (mL kg^−1^ min^−1^)	Before pre-20TT	49.5 [45.4, 53.5]	50.6 [46.0, 55.1]	–	–
Absolute $\dot{V}{\text{O}}_{{2{\text{peak}}}}$ (L min^−1^)	Before pre-20TT	3.99 [3.45, 4.53]	4.06 [3.46, 4.65]	–	–
Peak power output (Watts)	Before pre-20TT	345 [298, 392]	346 [300, 393]	–	–

Values reported as mean [95% confidence interval]

*CON* control, *HA* heat acclimation, *HR* heart rate, *T*_*re*_ rectal temperature, *PV* plasma volume, *I-FABP* intestinal fatty acid binding protein, *TNF-α* tumour necrosis factor alpha, *IL-6* interleukin 6, $\dot{V}{\text{O}}_{{2{\text{peak}}}}$ peak aerobic capacity

^a^Statistically different to CON at the same timepoint

#### Physiological measures

In the post-intervention time trial, *T*_*s*k_ was statistically higher in HA compared to CON from 18 km onwards (*p* = 0*.*037–0.039; *d* = 0.36–0.55). The HR, T_re_ and RPE during 20TT were not statistically different between conditions at any point (Supplement 2 – Output 4). The HR, *T*_re_ and *T*_sk_ and RPE responses during 20TT are shown in Supplement 3—Figs. 3 & 4.

### Training days 1 and 5

#### Neuromuscular measures

There was a *day* by *condition* effect on training day MVC (Supplement 2—Output 5). However, post hoc tests showed this two-way interaction effect was due to a within, rather than between, condition difference for HA. Voluntary activation and MVC were not statistically different between conditions at any point during training. Pre-intervention M-wave and post-exercise Pt values were statistically higher in HA compared to CON (both *p* < 0.001). Post-exercise RR on day one was statistically higher in HA than CON (*p* = 0*.*027; *d* = 1.67 [0.20, 3.14]). CD, RTD, 0.5RT, V-M_max_, EMG-M_max_ and V-wave showed no between condition differences at any point during training (Supplement 2—Output 5). See Supplement 4—Table 2 for neuromuscular data from training days.

#### Performance and physiological measures

Training day power output and cadence did not differ between conditions at any point (Supplement 2—Output 6). The HR in HA was statistically higher than CON after 25 min on training day one (*p* = 0*.*001–0.028; MD = 13 to 23 beats min^−1^ [2, 35]; *d* = 2.61–4.34), but only from 40 min onwards by day five (*p* = 0*.*012–0.038; MD = 13 to 17 beats min^−1^ [1, 29]; *d* = 2.44–3.12). The *T*_re_ on training day one was statistically higher in HA compared to CON (MD = 0.24 °C [0.09, 0.39]; *p* = 0.007; *d* = 1.46 [0.60, 2.31]), but not different on day five (*p* = 0*.6*64). The T_sk_ during training was statistically higher in HA compared to CON on days one and five at all timepoints (all *p* < 0.001; *d* = 10.5–15.4). The RPE was higher in HA than CON from 30 min onwards regardless of the day (all *p* < 0.001; MD = 1–2 AU 95% CI [0,2]; *δ*_Cliff_ = 0.29–0.49). The HR, *T*_re_ and *T*_sk_ and RPE responses during training days are shown in Supplement 3 – Fig. 5 & 6. Urine osmolality and urine specific gravity were statistically higher on day 5 than day 1 (*p* = 0.024–0.047; Supplement 2—Output 6), although there were no *condition* or *day* by *condition* effects.

## Discussion

This study investigated the effect of short-term HA on neuromuscular function and self-paced cycling performance in the heat by recreationally trained athletes. Contrasting our hypothesis, five days of moderate-intensity HA training did not statistically improve 20 km cycling performance time in the heat, compared to five days of training in a temperate environment. Interestingly, there was evidence that HA preserved knee extensor MVC torque compared to the control (Table 1). This increase is unlikely due to improved descending drive as V:M_max_, EMG:M_max_ and voluntary activation remained unchanged following HA. Accordingly, these data suggest that an early adaptation from short-term HA training could be improved knee extensor strength, potentially performance-enhancing for tasks, such as tactical operator load carriage (Orr et al. 2019) or vertical jump height (Pääsuke et al. 2001).

Previous studies have demonstrated performance benefits from HA training (Garrett et al. 2011; Chalmers et al. 2014; Périard et al. 2015), although considerable methodological variations (e.g., exercise mode, training duration, intensity, and environmental condition) confounds interpretation of aggregated results. In the context of short-term HA training, relatively few studies have used a self-paced performance task in the heat (Keiser et al. 2015; Racinais et al. 2015; Willmott et al. 2016). In the absence of a control group, Racinais et al. (2015) reported that highly trained cyclists improved cycling performance by 10.3% following five days of HA training. The performance change was ~ threefold greater than the current study (10.3% compared to 3.6%) and may be due to the longer duration of the performance task (43.4 km versus 20 km) and/or greater training duration (> 240 min day^−1^ versus 60 min day^−1^) (Racinais et al. 2015). The disparity in participant training status might also influence the observed difference in study outcomes (professional cyclists versus recreational athletes). Notably conflicting with this suggestion, previous evidence suggests that highly trained athletes may have less adaptive potential due to pre-existing training-induced acclimation status (Cheung and McLellan 1998; Garrett et al. 2011). A similar and comparable improvement (9.6%) in work completed in 30 min was seen following ten days of 90 min heat training (Keiser et al. 2015). However, participants were immersed in a hot water bath for 20 min before commencing the time trial, again challenging parallels with the present study’s outcomes.

In contrast to medium- and long-term HA training (typically ≥ 7 days), the reduced time requirements of short-term HA is appealing to many athletes and coaches (Racinais and Périard 2020). Crucially, the limited time course of short-term HA has reportedly facilitated partial physiological and perceptual adaptations to subsequent heat stress, such as plasma volume expansion (Périard et al. 2015). In the present study, the mean observed increase in plasma volume for HA (9.0%) aligns with previously reported values (4.2–9.8%) (Garrett et al. 2011). However, the lack of a reduction in resting heart rate suggests that the current protocol failed to induce HA adaptations, as moderate changes (− 5 ± 1 beats min^−1^) in resting heart rate are reported to occur rapidly, even during short-term HA training (Tyler et al. 2016). Similarly, the absence of a statistically discernible change in other heat adaptation measures (i.e., *T*_re_ and *T*_sk;_ Table 2) between conditions suggests that five days of moderate-intensity HA training may be insufficient to actuate these thermo-physiological enhancements in recreationally trained athletes. This evidence matches the findings of similar short-term HA protocols, which did not observe a statistical change in some of these physiological variables (Garrett et al. 2012; Willmott et al. 2017). Post-intervention 20TT sweat rate was found to be higher in HA than CON (*p* = 0.007; *d* = 1.82), despite previous research suggesting a prolonged timeframe for sweat rate adaptations, such as medium- and long-term HA training (Tyler et al. 2016). However, this difference possibly owes to the use of a self-paced performance task in the present study which permitted a higher workload to be completed in the post-intervention HA 20TT, than a true thermo-physiological adaptation in sweat rate due to acclimation. Although there was no statistically significant condition difference in post-intervention 20TT completion times, there was a trend towards a faster time in HA and, therefore, a higher resultant sweat rate (Table 2). The workload-matched training data provide further support for this hypothesis, as no differences in sweat rate were observed between the first and final HA training days (Supplement 2 Output 6), indicating no sweat response adaptations.

We hypothesised that adaptations arising from short-term HA training, such as an attenuation of intestinal damage and reduced expression of inflammatory cytokines, may attenuate central fatigue, maintain neuromuscular function and enhance exercise performance (Nybo and Nielsen 2001; Lambert 2004; Kuennen et al. 2011). No difference in the level of inflammatory cytokines (TNF-α and IL-6) or markers of gut damage (I-FABP) was seen between conditions (Table 2), suggesting that short-term HA may not protect against inflammatory heat stress during 20 km self-paced cycling in the heat. Following the HA intervention, knee extensor MVC torque increased, while descending input measures to the quadriceps motoneurons remained unchanged (Table 1; Supplement 4—Table 1). This could suggest that HA did not reduce central input to motoneurons during maximal contractions. Greater knee extensor torque after the HA intervention may be explained by increased input from supraspinal centres, peripheral inputs, or morphological changes within the muscle itself (Taylor et al. 2016). As no change was seen in any measures of descending drive input to quadriceps motoneurons (i.e., voluntary activation, EMG:M_max_ and V:M_max_), it seems that alterations in descending drive did not underpin the observed changes in MVC torque. Measures of peripheral nervous system function were not collected as this was not within the scope of the study. However, it is unlikely that changes in peripheral inputs to the quadriceps motoneurons capable of improving strength would occur following heat acclimation (Racinais et al. 2017a). Changes in muscle contractility are known to influence MVC torque, and although increased RTD and lower 0.5RT have been observed following passive HA (Racinais et al. 2017b), this was not seen in the present study and thus does not explain increases in knee extensor MVC torque. Although speculative, one explanation for the increases in knee extensor torque may have been due to increased muscle volume, as has been observed following passive heat acclimation (Rodrigues et al. 2020). However, as muscle volume was not measured in the present study, there is no conclusive evidence to suggest this is the mechanism underpinning the observed strength changes.

The HA performance change in the present study was not statistically different to CON (*p* = 0*.*076). Further examination of individual completion times (Fig. 1A) indicates considerable inter-participant performance variation in response to HA training, which is reflected in wide bounds of the mean completion time difference (− 9 to 145 s; Fig. 1B). The precise reason for the observed inter-individual variability in response to HA is difficult to determine. It could relate to an individualised genetic or phenotypic characteristics that modulate the magnitude of the response to HA adaptation (Taylor 2014; Corbett et al. 2018; Alkemade et al. 2021). Conceivably, some athletes or coaches may consider short-term HA training an acceptable risk to obtain a possible performance advantage. However, as previously discussed, the limited adaptive potential of more highly trained athletes could result in reduced performance benefits (Garrett et al. 2011). Hence, the risk of compromised training quality may outweigh a possible performance improvement.

A limitation of the present study was the small sample size and the use of recreationally trained participants, limiting the results’ applicability to a more highly trained cohort. It is unlikely that recreational athletes would actively choose to undertake heat acclimation training compared to elite competitive athletes. However, the lack of a clear performance improvement in the present study, despite the arguably greater adaptive potential of a recreationally trained cohort (Garrett et al. 2011), may suggest that highly trained athletes avoid moderate-intensity short-term HA training due to limited beneficial performance outcomes. As previously discussed, the 20TT used in the present study was selected to assess the effect of HA training on a self-paced performance task. However, the self-paced nature of the 20TT, with associated fluctuations in exercise intensity, made it challenging to identify the effect of HA adaptations of measured variables. Utilising a fixed-load task before the 20TT may have provided additional information around these changes. There is also the possibility of an ordering effect on the second training block and performance trials. However, this is unlikely as participants were assigned in a randomised and counter-balanced order, with at least 34 days between conditions, and no significant differences were identified in pre-trial measures of aerobic capacity (i.e., $\dot{V}{\text{O}}_{{2{\text{peak}}}}$ or peak power output).

## Conclusion

This study shows that five days of moderate-intensity HA training by recreationally trained athletes does not improve 20 km self-paced cycling performance in the heat. Further, this intervention may not confer many of the traditional training adaptations observed in longer HA protocols when compared to a workload-matched control environment. The higher MVC following HA than CON is novel, yet requires additional consideration given the similarities in voluntary activation and other neuromuscular properties. Finally, conflicting with the hypothesis, HA did not protect against inflammatory cytokines release or intestinal damage after cycling performance in hot conditions. Interpretation of these findings could suggest that when time and resources are limited, the maintenance of training quality in a temperate environment before an athletic competition in the heat should be of higher priority than undertaking a short-term fixed-intensity heat-based training program.

## Supplementary Information

Below is the link to the electronic supplementary material.

Supplementary file1 (PDF 130 kb)

Supplementary file2 (PDF 407 kb)

Supplementary file3 (TIFF 35160 kb)

Supplementary file4 (TIFF 114 kb)

Supplementary file5 (TIFF 403 kb)

Supplementary file6 (TIFF 9846 kb)

Supplementary file7 (TIFF 358 kb)

Supplementary file8 (TIFF 113 kb)

Supplementary file9 (DOCX 24 kb)

## Abbreviations

½ RT: Half relaxation time (ms)
20TT: 20 km time trial
CD: Contraction duration (ms)
CI: Confidence interval
CON: Control
CV: Coefficient of variation
ELISA: Enzyme-linked immunosorbent assay
EMG: Electromyography
HA: Heat acclimation
HR: Heart rate (beats min^−^^1^)
ICC: Intraclass correlation
I-FABP: Intestinal fatty acid binding protein (pg mL^−^^1^)
IL-6: Interleukin 6 (pg mL^−^^1^)
MD: Mean difference
MVC: Maximum voluntary contraction (N m)
ms: Milliseconds
PPO: Peak power output (Watts)
Pt: Potentiated twitch (N m)
PV: Plasma volume (%)
RH: Relative humidity (%)
RPE: Rating of perceived exertion
RR: Rate of relaxation (N m s^−^^1^)
RTD: Rate of torque development (N m s^−^^1^)
SD: Standard deviation
*T*_re_: Core (rectal) temperature (°C)
TNF-α: Tumour necrosis factor alpha (pg mL^−^^1^)
TPt: Time to peak torque (ms)
*T*_sk_: Mean skin temperature (°C)
VA: Voluntary activation (%)
$\dot{V}{\text{O}}_{{2{\text{peak}}}}$: Peak aerobic capacity (mL kg^−1^ min^−1^)

## References

[CR1] Alkemade P, Gerrett N, Eijsvogels TMH, Daanen HAM (2021). Individual characteristics associated with the magnitude of heat acclimation adaptations. Eur J Appl Physiol.

[CR2] Allen GM, Gandevia SC, McKenzie DK (1995). Reliability of measurements of muscle strength and voluntary activation using twitch interpolation. Muscle Nerve.

[CR3] Ashley CD, Ferron J, Bernard TE (2015). Loss of heat acclimation and time to re-establish acclimation. J Occup Environ Hyg.

[CR4] Benjamin CL, Sekiguchi Y, Fry LA, Casa DJ (2019). Performance changes following heat acclimation and the factors that influence these changes: meta-analysis and meta-regression. Front Physiol.

[CR5] Borg G (1998). Borg’s perceived exertion and pain scales.

[CR6] Borg DN, Osborne JO, Stewart IB (2018). The reproducibility of 10 and 20km time trial cycling performance in recreational cyclists, runners and team sport athletes. J Sci Med Sport.

[CR7] Brade C, Dawson B, Wallman K (2013). Effect of precooling and acclimation on repeat-sprint performance in heat. J Sports Sci.

[CR8] Brazaitis M, Skurvydas A (2010). Heat acclimation does not reduce the impact of hyperthermia on central fatigue. Eur J Appl Physiol.

[CR9] Chalmers S, Esterman A, Eston R (2014). Short-term heat acclimation training improves physical performance: a systematic review, and exploration of physiological adaptations and application for team sports. Sports Med.

[CR10] Chen T-I, Tsai P-H, Lin J-H (2013). Effect of short-term heat acclimation on endurance time and skin blood flow in trained athletes. Open Access J Sports Med.

[CR11] Cheung SS (2007). Hyperthermia and voluntary exhaustion: integrating models and future challenges. Appl Physiol Nutr Metab.

[CR12] Cheung SS, McLellan TM (1998). Heat acclimation, aerobic fitness, and hydration effects on tolerance during uncompensable heat stress. J Appl Physiol.

[CR13] Cliff N (1996). Answering ordinal questions with ordinal data using ordinal statistics. Multivar Behav Res.

[CR14] Cohen J (1992). A power primer. Psychol Bull.

[CR15] Corbett J, Rendell RA, Massey HC (2018). Inter-individual variation in the adaptive response to heat acclimation. J Therm Biol.

[CR16] Currell K, Jeukendrup AE (2008). Validity, reliability and sensitivity of measures of sporting performance. Sports Med.

[CR17] Daanen HAM, Racinais S, Périard JD (2018). Heat acclimation decay and re-induction: a systematic review and meta-analysis. Sports Med.

[CR18] Davis JM, Bailey SP (1997). Possible mechanisms of central nervous system fatigue during exercise. Med Sci Sports Exerc.

[CR19] De Pauw K, Roelands B, Cheung SS (2013). Guidelines to classify subject groups in sport-science research. Int J Sports Physiol Perform.

[CR20] Dill DB, Costill DL (1974). Calculation of percentage changes in volumes of blood, plasma, and red cells in dehydration. J Appl Physiol.

[CR21] Garrett AT, Goosens NG, Rehrer NG (2009). Induction and decay of short-term heat acclimation. Eur J Appl Physiol.

[CR22] Garrett AT, Rehrer NJ, Patterson MJ (2011). Induction and decay of short-term heat acclimation in moderately and highly trained athletes. Sports Med.

[CR23] Garrett AT, Creasy R, Rehrer NJ (2012). Effectiveness of short-term heat acclimation for highly trained athletes. Eur J Appl Physiol.

[CR24] Garrett AT, Dodd E, Biddlecombe V (2019). Effectiveness of short-term heat acclimation on intermittent sprint performance with moderately trained females controlling for menstrual cycle phase. Front Physiol.

[CR25] Girard O, Bishop DJ, Racinais S (2018). M-wave normalization of EMG signal to investigate heat stress and fatigue. J Sci Med Sport.

[CR26] ISO 9886 (2004). Evaluation of thermal strain by physiological measurements.

[CR27] Keiser S, Flück D, Hüppin F (2015). Heat training increases exercise capacity in hot but not in temperate conditions: a mechanistic counter-balanced cross-over study. Am J Physiol - Heart Circ Physiol.

[CR28] Kuennen M, Gillum T, Dokladny K (2011). Thermotolerance and heat acclimation may share a common mechanism in humans. Am J Physiol - Regul Integr Comp Physiol.

[CR29] Lakens D (2017). Equivalence tests: a practical primer for t tests, correlations, and meta-analyses. Soc Psychol Personal Sci.

[CR30] Lambert GP (2004). Role of gastrointestinal permeability in exertional heatstroke. Exerc Sport Sci Rev.

[CR31] Lambert GP (2008). Intestinal barrier dysfunction, endotoxemia, and gastrointestinal symptoms: the “canary in the coal mine” during exercise-heat stress?. Med Sport Sci.

[CR32] Lee BJ, Miller A, James RS, Thake CD (2016). Cross acclimation between heat and hypoxia: heat acclimation improves cellular tolerance and exercise performance in acute normobaric hypoxia. Front Physiol.

[CR33] Lim CL, Mackinnon LT (2006). The roles of exercise-induced immune system disturbances in the pathology of heat stroke: the dual pathway model of heat stroke. Sports Med.

[CR34] Marino FE (2010). The limitations of the constant load and self-paced exercise models of exercise physiology. Comp Exerc Physiol.

[CR35] Moseley PL, Gapen C, Wallen ES (1994). Thermal stress induces epithelial permeability. Am J Physiol Cell Physiol.

[CR36] Nava R, Zuhl MN (2020). Heat acclimation-induced intracellular HSP70 in humans: a meta-analysis. Cell Stress Chaperones.

[CR37] Nybo L, González-Alonso J (2015). Critical core temperature: a hypothesis too simplistic to explain hyperthermia-induced fatigue. Scand J Med Sci Sports.

[CR38] Nybo L, Nielsen B (2001). Hyperthermia and central fatigue during prolonged exercise in humans. J Appl Physiol.

[CR39] Nybo L, Rasmussen P, Sawka MN (2014). Performance in the heat-physiological factors of importance for hyperthermia-induced fatigue. Compr Physiol.

[CR40] Orr RM, Dawes JJ, Lockie RG, Godeassi DP (2019). The relationship between lower-body strength and power, and load carriage tasks: a critical review. Int J Exerc Sci.

[CR41] Osborne JO, Stewart IB, Beagley KW (2019). Acute glutamine supplementation does not improve 20-km self-paced cycling performance in the heat. Eur J Appl Physiol.

[CR42] Osborne JO, Stewart IB, Beagley KW, Minett GM (2019). The effect of cycling in the heat on gastrointestinal-induced damage and neuromuscular fatigue. Eur J Appl Physiol.

[CR43] Pääsuke M, Ereline J, Gapeyeva H (2001). Knee extension strength and vertical jumping performance in nordic combined athletes. J Sports Med Phys Fitness.

[CR44] Périard JD, Racinais S, Sawka MN (2015). Adaptations and mechanisms of human heat acclimation: applications for competitive athletes and sports. Scand J Med Sci Sports.

[CR45] R Core Team (2020) R: a language and environment for statistical computing. Version 4.0.2. R Foundation for Statistical Computing, Vienna, Austria. https://www.R-project.org

[CR46] Racinais S, Périard JD (2020). Benefits of heat re-acclimation in the lead-up to the Tokyo Olympics. Br J Sports Med.

[CR47] Racinais S, Périard JD, Karlsen A, Nybo L (2015). Effect of heat and heat acclimatization on cycling time trial performance and pacing. Med Sci Sports Exerc.

[CR48] Racinais S, Wilson MG, Gaoua N, Périard JD (2017). Heat acclimation has a protective effect on the central but not peripheral nervous system. J Appl Physiol.

[CR49] Racinais S, Wilson MG, Périard JD (2017). Passive heat acclimation improves skeletal muscle contractility in humans. Am J Physiol Regul Integr Comp Physiol.

[CR50] Rodrigues P, Trajano GS, Wharton L, Minett GM (2020). Effects of passive heating intervention on muscle hypertrophy and neuromuscular function: a preliminary systematic review with meta-analysis. J Therm Biol.

[CR51] Romano J, Kromrey JD, Coraggio J, Skowronek J (2006) Appropriate statistics for ordinal level data: Should we really be using t-test and Cohen’sd for evaluating group differences on the NSSE and other surveys? In: Annual Meeting of the Florida Association of Institutional Research. pp 1–3

[CR52] Sawka MN, Leon LR, Montain SJ, Sonna LA (2011). Integrated physiological mechanisms of exercise performance, adaptation, and maladaptation to heat stress. Compr Physiol.

[CR53] Sunderland C, Morris JG, Nevill ME (2008). A heat acclimation protocol for team sports. Br J Sports Med.

[CR54] Taylor NA (2014). Human heat adaptation. Compr Physiol.

[CR55] Taylor JL, Amann M, Duchateau J (2016). Neural contributions to muscle fatigue: from the brain to the muscle and back again. Med Sci Sports Exerc.

[CR56] Tucker R, Noakes TD (2009). The physiological regulation of pacing strategy during exercise: a critical review. Br J Sports Med.

[CR57] Tyler CJ, Reeve T, Hodges GJ, Cheung SS (2016). The effects of heat adaptation on physiology, perception and exercise performance in the heat: a meta-analysis. Sports Med Auckl NZ.

[CR58] Vargas NT, Marino F (2014). A neuroinflammatory model for acute fatigue during exercise. Sports Med.

[CR59] Wickham H (2016). ggplot2: elegant graphics for data analysis.

[CR60] Willmott AGB, Gibson OR, Hayes M, Maxwell NS (2016). The effects of single versus twice daily short term heat acclimation on heat strain and 3000m running performance in hot, humid conditions. J Therm Biol.

[CR61] Willmott AGB, Hayes M, Waldock KAM (2017). Short-term heat acclimation prior to a multi-day desert ultra-marathon improves physiological and psychological responses without compromising immune status. J Sports Sci.

[CR62] Wingfield GL, Gale R, Minett GM (2016). The effect of high versus low intensity heat acclimation on performance and neuromuscular responses. J Therm Biol.

