# Supplementary material for: Short-term heat acclimation preserves knee extensor torque but does not improve 20 km self-paced cycling performance in the heat
Source: Eur J Appl Physiol. 2021 Jun 19;121(10):2761–72. doi: 10.1007/s00421-021-04744-y (PMC8416835; doi:10.1007/s00421-021-04744-y)
Supplement: Supplementary file 1 — Supplementary file1 (PDF 130 kb) [file 421_2021_4744_MOESM1_ESM.pdf]

Supplement 1 – R Packages and Power Analysis Code

R Package List.

1. R Core Team. *R: A Language and Environment for Statistical Computing*. R Foundation for Statistical Computing; 2020. https://www.R-project.org

2. Lakens D. *TOSTER: Two One-Sided Tests (TOST) Equivalence Testing*.; 2018. Accessed September 28, 2020. https://CRAN.R-project.org/package=TOSTER

3. Tierney N, Cook D, McBain M, et al. *Naniar: Data Structures, Summaries, and Visualisations for Missing Data*.; 2020. Accessed September 28, 2020. https://CRAN.R-project.org/package=naniar

4. Kuznetsova A, Brockhoff PB, Christensen RHB, Jensen SP. *LmerTest: Tests in Linear Mixed Effects Models*.; 2020. Accessed September 28, 2020. https://CRAN.R-project.org/package=lmerTest

5. Lenth R, Buerkner P, Herve M, Love J, Riebl H, Singmann H. *Emmeans: Estimated Marginal Means, Aka Least-Squares Means*.; 2020. Accessed September 28, 2020. https://CRAN.R-project.org/package=emmeans

6. Torchiano M. *Effsize: Efficient Effect Size Computation*.; 2020. Accessed September 28, 2020. https://CRAN.R-project.org/package=effsize

7. Wickham H. *Ggplot2: Elegant Graphics for Data Analysis*. Springer; 2016.

Power Analysis Output

```
#Load packages
library(simr)

## Loading required package: lme4

## Loading required package: Matrix

## Registered S3 methods overwritten by 'car':
##   method                          from
##   influence.merMod                lme4
##   cooks.distance.influence.merMod lme4
##   dfbeta.influence.merMod         lme4
##   dfbetas.influence.merMod        lme4

##
## Attaching package: 'simr'

## The following object is masked from 'package:lme4':
##
##     getData

# Create dataset with participants (ID), condition and test
ID <- 1:8
condition <- letters[1:2]
```

```
test <- letters[1:2]
data <- expand.grid(ID = ID, condition = condition, test = test)

# Specify fixed and random parameters
# Intercept - (2169) taken from team sport 20TT time from Borg (2018).
# Condition - (0) No  difference at baseline as repeated measures design.
# Test - (-15) average improvement (0.7%) in the CON training groups of th
e following HA studies: Lee (0.8% - 10days HA); Wilmott (0.6%)
# Condition*Test - average HA improvement (4.5%) of Wingfield (5.9%); Lee
(4.8% - 10days); Wilmott (2.8%)) minus the test effect (0.7%) = 3.8%;83 se
conds.

b <- c(2169, 0, -15, -83)
s <- 40 # Residual standard deviation


# Random intercept
sd_random_inter <- 99 #from SD of 20TT completion time of team sport athle
tes in Borg (2018)
V_mat = sd_random_inter^2

# Model
model1 <- makeLmer(y ~ condition*test + (1|ID), fixef = b, VarCorr = V_mat
, sigma = s, data = data)
print(model1)

## Linear mixed model fit by REML ['lmerMod']
## Formula: y ~ condition * test + (1 | ID)
##    Data: data
## REML criterion at convergence: 495.6249
## Random effects:
##  Groups   Name        Std.Dev.
##  ID       (Intercept) 99
##  Residual             40
## Number of obs: 32, groups:  ID, 8
## Fixed Effects:
##      (Intercept)        conditionb             testb  conditionb:testb
##             2169                 0               -15               -83

# Simulation
powerSim(model1, test = fixed("condition:test"), nsim = 1000)

## Power for predictor 'condition:test', (95% confidence interval):
##       83.20% (79.63, 86.37)
##
## Test: Kenward Roger (package pbkrtest)
##
## Based on 1000 simulations, (0 warnings, 0 errors)
## alpha = 0.05, nrow = 32
##
## Time elapsed: 0 h 0 m 41 s
```
