# Supplementary material for: Short-term heat acclimation preserves knee extensor torque but does not improve 20 km self-paced cycling performance in the heat
Source: Eur J Appl Physiol. 2021 Jun 19;121(10):2761–72. doi: 10.1007/s00421-021-04744-y (PMC8416835; doi:10.1007/s00421-021-04744-y)
Supplement: Supplementary file 2 — Supplementary file2 (PDF 407 kb) [file 421_2021_4744_MOESM2_ESM.pdf]

Supplement 2 - Model Outputs

Output 1. Time trial performance variables.

	**Variable**			
	**Completion time (seconds)**			
Parameter	β	SE	95% CI	p-value
Intercept	2226.88	71.91	2078.98 - 2374.77	< .001*
Condition	-30.50	26.73	81.53 - 20.53	.267
Test	-41.25	26.73	-92.28 - 9.78	.138
Condition by Test	-37.25	37.80	-109.42 - 34.92	.336
	**Power difference [Test 2 – Test 1] (Watts)**			
Parameter	β	SE	95% CI	p-value
Intercept	6.07	6.96	-7.58 – 19.72	.401
Test	9.51	7.33	-4.86 – 23.87	.233
KM^1	23.79	164.44	-298.51 – 346.08	.887
KM^2	-16.62	82.32	-177.96 – 144.73	.843
Test by KM^1	40.08	209.84	-371.21 – 451.37	.854
Test by KM^2	77.74	102.22	-122.61 – 278.08	.465
	**Cadence difference [Test 2 – Test 1] (revolutions·min^{-1})**			
Parameter	β	SE	95% CI	p-value
Intercept	2.25	4.48	-6.53 – 11.04	.624
Test	-1.13	2.78	-6.58 – 4.33	.694
KM^1	-51.88	37.66	-125.70 – 21.93	.202
KM^2	-51.88	29.57	-109.83 – 6.06	.126
Test by KM^1	27.62	21.86	-15.23 – 70.47	.254
Test by KM^2	37.34	17.82	2.41 – 72.26	.097
	**Mean power (Watts)**			
Parameter	β	SE	95% CI	p-value
Intercept	197.27	19.39	159.26 – 235.29	<.001*
Condition	5.80	6.06	-6.08 – 17.68	.349
Test	7.66	6.06	-4.21 – 19.54	.220
Condition by Test	9.92	8.57	-6.87 – 26.72	.260
	**Mean cadence (revolutions·min^{-1})**			
Parameter	β	SE	95% CI	p-value
Intercept	92.12	3.34	85.58 – 98.67	<.001*
Condition	1.00	2.09	-3.10 – 5.10	.638
Test	-0.50	2.09	-4.60 – 3.60	.813
Condition by Test	-1.00	2.96	-6.80 – 4.80	.739

* Statistically important effect.
SE = standard error; CI = confidence interval.
Condition = HA or CON; Test = pre- or post-5 day training intervention; KM = distance of 20TT in km.

Output 2. Neuromuscular variables before and after time trial performance.

	**Variable**			
	**Maximum voluntary contraction [MVC] (N·m)**			
Parameter	β	SE	95% CI	p-value
Intercept	254.95	13.11	229.25 – 280.65	<.001*
Time	-36.16	6.04	-48.00 – -24.32	<.001*
Test	-14.69	5.74	-25.95 – -3.43	.011*
Condition	-9.58	5.55	-20.46 – 1.30	.086
Time by Test	21.54	8.42	5.04 – 38.05	.011*
Time by Condition	7.10	8.16	-8.89 – 23.09	.386
Test by Condition	27.45	8.08	11.62 – 43.28	.001*
Time by Test by Condition	-13.59	11.56	-36.24 – 9.05	.241
	**Voluntary activation [VA] (%)**			
Parameter	β	SE	95% CI	p-value
Intercept	92.64	1.74	89.22 – 96.06	<.001*
Time	-5.85	1.50	-8.80 – -2.91	<.001*
Test	0.19	1.45	-2.65 – 3.02	.898
Condition	0.22	1.38	-2.49 – 2.92	.875
Time by Test	3.54	2.11	-0.59 – 7.67	.095
Time by Condition	-1.43	2.04	-5.43 – 2.57	.484
Test by Condition	0.64	2.02	-3.32 – 4.61	.751
Time by Test by Condition	-0.13	2.89	-5.80 – 5.54	.965
	**Potentiated twitch [Pt] (N·m)**			
Parameter	β	SE	95% CI	p-value
Intercept	67.38	3.84	59.84 – 74.91	<.001*
Time	-19.11	2.30	-23.61 – -14.61	<.001*
Test	-0.68	2.21	-5.02 – 3.66	.759
Condition	1.43	2.11	-2.71 – 5.56	.500
Time by Test	0.81	3.22	-5.50 – 7.13	.801
Time by Condition	-0.52	3.12	-6.64 – 5.60	.867
Test by Condition	-2.72	3.09	-8.79 – 3.34	.380
Time by Test by Condition	-1.37	4.42	-10.04 – 7.29	.757
	**Contraction duration (CD) (ms)**			
Parameter	β	SE	95% CI	p-value
Intercept	149.77	7.32	135.43 – 164.11	<.001*
Time	-33.14	3.07	-39.17 – -27.12	<.001*
Test	0.55	3.03	-5.40 – 6.50	.857
Condition	-1.20	5.15	-11.30 – 8.91	.821
Time by Test	-2.22	4.42	-10.89 – 6.45	.616
Time by Condition	-1.50	4.17	-9.68 – 6.68	.720
Test by Condition	-1.45	4.11	-9.49 – 6.60	.725
Time by Test by Condition	0.40	5.94	-11.24 – 12.04	.947
	**Rate of torque development [RTD] (N·m·s^{-1})**			
Parameter	β	SE	95% CI	p-value
Intercept	844.62	0.08	729.02 – 978.55	<.001*
Time	0.80	0.04	0.74 – 0.87	<.001*
Test	0.97	0.04	0.90 – 1.06	.549
Condition	0.96	0.07	0.84 – 1.10	.592
Time by Test	1.06	0.06	0.94 – 1.20	.316
Time by Condition	1.11	0.06	1.00 – 1.25	.061
Test by Condition	1.08	0.06	0.97 – 1.21	.178
Time by Test by Condition	0.88	0.08	0.75 – 1.04	.134
	**Rate of Relaxation (RR) (N·m·s^{-1})**			
Parameter	β	SE	95% CI	p-value
Intercept	543.14	62.33	420.98 – 665.30	<.001*
Time	6.72	28.45	-49.03 – 62.47	.814
Test	-63.14	28.83	-119.64 – -6.64	.030*
Condition	-10.47	55.10	-118.45 – 97.51	.853
Time by Test	54.79	41.30	-26.16 – 135.73	.187
Time by Condition	10.65	38.60	-65.00 – 86.31	.783

Test by Condition	0.76	38.53	-74.77 – 76.29	.984
Time by Test by Condition	-24.99	55.21	-133.20 – 83.22	.651
	**Half relaxation time [0.5RT] (ms)**			
Parameter	β	SE	95% CI	p-value
Intercept	69.72	6.67	56.64 – 82.79	<.001*
Time	-21.71	2.31	-26.24 – -17.18	<.001*
Test	3.27	2.34	-1.32 – 7.86	.165
Condition	-4.75	4.62	-13.80 – 4.31	.330
Time by Test	-3.65	3.35	-10.22 – 2.93	.279
Time by Condition	4.05	3.14	-2.10 – 10.20	.198
Test by Condition	2.90	3.13	-3.23 – 9.04	.356
Time by Test by Condition	-3.25	4.49	-12.04 – 5.54	.470
	**V-M_{max}**			
Parameter	β	SE	95% CI	p-value
Intercept	0.26	0.04	0.18 – 0.34	<.001*
Time	-0.02	0.03	-0.09 – 0.04	.496
Test	0.01	0.03	-0.05 – 0.08	.634
Condition	0.07	0.03	0.01 – 0.13	.025*
Time by Test	-0.05	0.05	-0.14 – 0.04	.285
Time by Condition	-0.07	0.05	-0.16 – 0.02	.119
Test by Condition	-0.03	0.04	-0.11 – 0.05	.461
Time by Test by Condition	0.07	0.07	-0.06 – 0.20	.313
	**EMG-M_{max}**			
Parameter	β	SE	95% CI	p-value
Intercept	61.85	0.09	52.10 – 73.41	<.001*
Time	0.84	0.08	0.72 – 0.98	.032*
Test	0.94	0.08	0.81 – 1.10	.470
Condition	0.79	0.08	0.68 – 0.92	.004*
Time by Test	1.06	0.11	0.85 – 1.32	.620
Time by Condition	1.22	0.11	0.98 – 1.52	.078
Test by Condition	1.46	0.11	1.17 – 1.81	.001*
Time by Test by Condition	0.70	0.16	0.51 – 0.95	.026*
	**M-wave (mV)**			
Parameter	β	SE	95% CI	p-value
Intercept	7.66	0.61	6.46 – 8.85	<.001*
Time	-1.94	0.47	-2.86 – -1.02	<.001*
Test	-2.02	0.43	-2.86 – -1.18	<.001*
Condition	0.83	0.87	-0.89 – 2.54	.374
Time by Test	1.60	0.65	0.33 – 2.88	.015*
Time by Condition	-0.56	0.65	-1.84 – 0.71	.389
Test by Condition	0.07	0.59	-1.08 – 1.22	.903
Time by Test by Condition	-0.23	0.91	-2.01 – 1.55	.801
	**V-wave (mV)**			
Parameter	β	SE	95% CI	p-value
Intercept	1.72	0.16	0.23 – 0.85	.007*
Time	0.68	0.15	-0.68 – -0.09	.011*
Test	0.77	0.14	-0.54 – 0.01	.061
Condition	1.41	0.21	-0.08 – 0.77	.141
Time by Test	0.95	0.21	-0.47 – 0.37	.819
Time by Condition	0.60	0.21	-0.91 – -0.09	.017*
Test by Condition	0.88	0.19	-0.50 – 0.25	.503
Time by Test by Condition	1.73	0.30	-0.03 – 1.13	.066

* Statistically important effect. SE = standard error; CI = confidence interval; EMG = electromyography. Time = before or after a 20TT; Test = pre- or post-5 day training intervention; Condition = HA or CON; KM = distance of 20TT in km.

Output 3. Time trial baseline and pre-post data.

	**Variable**			
	**Baseline HR (beats·min^{-1})**			
Parameter	β	SE	95% CI	p-value
Intercept	54.95	2.40	50.25 – 59.66	<.001*
Condition	-2.35	2.30	-6.86 – 2.16	.319
Test	-4.61	2.30	-9.12 – -0.11	.058
Condition by Test	1.72	3.32	-4.79 – 8.24	.610
	**Baseline T_{re} (°C)**			
Parameter	β	SE	95% CI	p-value
Intercept	37.04	0.08	36.89 – 37.19	<.001*
Condition	0.14	0.08	-0.03 – 0.30	.114
Test	0.03	0.08	-0.13 – 0.19	.734
Condition by Test	-0.08	0.12	-0.31 – 0.15	.512
	**I-FABP (pg·mL^{-1})**			
Parameter	β	SE	95% CI	p-value
Intercept	561.34	125.21	315.95 – 806.74	<.001*
Time	372.76	158.12	62.86 – 682.67	.023*
Test	134.90	163.58	-185.70 – 455.51	.414
Condition	-109.84	152.88	-409.47 – 189.79	.476
Time by Test	303.74	219.20	-125.89 – 733.37	.173
Time by Condition	150.50	216.03	-272.91 – 573.91	.490
Test by Condition	94.47	218.41	-333.60 – 522.55	.668
Time by Test by Condition	-90.01	300.61	-679.18 – 499.17	.766
	**TNF-α (pg·mL^{-1})**			
Parameter	β	SE	95% CI	p-value
Intercept	3.47	0.66	2.18 – 4.77	<.001*
Time	1.41	0.78	-0.13 – 2.94	.080
Test	-0.10	0.78	-1.64 – 1.44	.899
Condition	0.15	0.73	-1.28 – 1.58	.836
Time by Test	0.84	1.09	-1.30 – 2.99	.446
Time by Condition	-0.59	1.07	-2.69 – 1.51	.583
Test by Condition	0.11	1.05	-1.95 – 2.17	.918
Time by Test by Condition	-1.70	1.50	-4.64 – 1.23	.262
	**IL-6 (pg·mL^{-1})**			
Parameter	β	SE	95% CI	p-value
Intercept	2.82	0.25	1.73 – 4.59	.001*
Test	0.70	0.28	0.41 – 1.20	.215
Condition	1.75	0.29	0.99 – 3.10	.070
Test by Condition	0.99	0.40	0.45 – 2.18	.985
	**Serum osmolality (mOsm·kg^{-1})**			
Parameter	β	SE	95% CI	p-value
Intercept	0.29	0.00	0.29 – 0.30	<.001*
Test	0.00	0.00	-0.00 – 0.00	.485
Condition	-0.00	0.00	-0.00 – 0.00	.657
Test by Condition	-0.00	0.00	-0.01 – 0.00	.628
	**Urine osmolality (mOsm·kg^{-1})**			
Parameter	β	SE	95% CI	p-value
Intercept	776	168	471 - 1095	<.001*
Test	-366	223	-789 - 61	.162
Condition	-274	219	-665 - 154	.246
Test by Condition	-288	308	-265 - 896	388
	**Urine specific gravity (AU)**			
Parameter	β	SE	95% CI	p-value
Intercept	1.01	0.00	0.00 – 0.00	<.001*
Test	-0.00	0.01	-0.01 – 0.00	.354
Condition	-0.00	0.01	-0.01 – 0.01	.635
Test by Condition	0.00	0.01	-0.01 – 0.01	.976
	**Body mass (kg)**			

Parameter	β	SE	95% CI	p-value
Intercept	80.18	0.05	72.04 – 89.24	<.001*
Test	1.00	0.01	0.98 – 1.01	.695
Condition	0.99	0.01	0.97 – 1.00	.091
Test by Condition	1.01	0.01	0.99 – 1.03	.404
	**Sweat rate (L·h^{-1})**			
Parameter	β	SE	95% CI	p-value
Intercept	-1.29	0.14	-1.56 – -1.02	<.001*
Test	0.02	0.11	-0.19 – 0.24	.841
Condition	0.10	0.12	-0.13 – 0.32	.421
Test by Condition	-0.50	0.17	-0.83 – -0.16	.010*

* Statistically important effect.

SE = standard error; CI = confidence interval; HR = heart rate; T_{re} = rectal temperature; I-FABP = intestinal fatty acid binding protein; TNF-α = tumour necrosis factor alpha; IL-6 = interleukin 6.

Time = before or after a 20TT; Condition = HA or CON; Test = pre- or post-5 day training intervention.

Output 4. Time trial physiological and perceptual variables.

	**Variable**			
	**HR (beats·min^{-1})**			
Parameter	β	SE	95% CI	p-value
Intercept	161.31	3.96	153.54 – 169.07	<.001*
KM^1	313.75	37.05	241.14 – 386.36	<.001*
KM^2	-39.54	12.19	-63.43 – -15.65	.001*
KM^3	104.02	12.19	80.13 – 127.91	<.001*
Test	-0.54	0.48	-1.49 – 0.40	.259
Condition	4.51	5.60	-6.47 – 15.48	.435
KM^1 by Test	47.77	17.24	13.98 – 81.56	.006*
KM^2 by Test	-15.15	17.24	-48.94 – 18.63	.380
KM^3 by Test	-9.44	17.24	-43.23 – 24.34	.584
KM^1 by Condition	-29.34	52.60	-132.43 – 73.74	.585
KM^2 by Condition	-41.92	17.72	-76.66 – -7.19	.018*
KM^3 by Condition	9.14	17.74	-25.62 – 43.90	.607
Test by Condition	-0.63	0.69	-1.99 – 0.73	.362
KM^1 by Test by Condition	3.97	24.82	-44.67 – 52.61	.873
KM^2 by Test by Condition	61.42	24.72	12.96 – 109.88	.013*
KM^3 by Test by Condition	-25.24	24.73	-73.71 – 23.24	.30
	**T_{re} (°C)**			
Parameter	β	SE	95% CI	p-value
Intercept	36.94	0.10	36.72 – 37.16	<.001*
KM	0.08	0.00	0.08 – 0.08	<.001*
Test	-0.02	0.04	-0.10 – 0.05	.500
Condition	0.10	0.12	-0.14 – 0.34	.405
KM by Test	-0.00	0.00	-0.01 – 0.00	.175
KM by Condition	-0.00	0.00	-0.01 – 0.01	.958
Test by Condition	-0.03	0.05	-0.14 – 0.07	.504
KM by Test by Condition	0.00	0.00	-0.00 – 0.01	.177
	**T_{sk} (°C)**			
Parameter	β	SE	95% CI	p-value
Intercept	35.91	0.12	35.67 – 36.15	<.001*
KM^1	22.10	1.87	18.43 – 25.77	<.001*
KM^2	-9.59	0.43	-10.43 – -8.76	<.001*
KM^3	2.51	0.42	1.68 – 3.34	<.001*
Test	-0.15	0.02	-0.19 – -0.12	<.001*
Condition	0.09	0.12	-0.15 – 0.33	.491
KM^1 by Test	-0.77	0.60	-1.94 – 0.41	.200
KM^2 by Test	-0.23	0.60	-1.41 – 0.94	.696
KM^3 by Test	1.00	0.59	-0.16 – 2.16	.093
KM^1 by Condition	-2.49	1.63	-5.69 – 0.70	.149
KM^2 by Condition	0.84	0.62	-0.36 – 2.05	.170
KM^3 by Condition	0.65	0.61	-0.55 – 1.85	.290
Test by Condition	0.06	0.02	0.01 – 0.11	.015*
KM^1 by Test by Condition	4.53	0.87	2.84 – 6.23	<.001*
KM^2 by Test by Condition	0.06	0.85	-1.62 – 1.73	.946
KM^3 by Test by Condition	-1.38	0.85	-3.04 – 0.28	.104
	**RPE (AU) [analysed using beta regression]**			
Parameter	β	SE	95% CI	p-value
Intercept	1.04	0.12	0.82 – 1.30	.760
Test	1.12	0.17	0.81 – 1.56	.490
KM	1.07	0.01	1.05 – 1.09	<.001*
Condition	0.87	0.17	0.63 – 1.21	.416
Test by KM	1.00	0.01	0.97 – 1.02	.781
Test by Condition	1.04	0.23	0.65 – 1.64	.879
KM by Condition	1.01	0.01	0.99 – 1.04	.335
Test by KM by Condition	0.99	0.02	0.95 – 1.02	.458

* Statistically important effect.
SE = standard error; CI = confidence interval; HR = heart rate; T_{re} = rectal temperature; T_{sk} = skin temperature; RPE = rating of perceived exertion.
KM = distance of 20TT in km; Test = pre- or post-5 day training intervention; Condition = HA or CON.

Output 5. Neuromuscular variables before and after training days 1 and 5.

	**Variable**			
	**Maximum voluntary contraction [MVC] (N·m)**			
Parameter	β	SE	95% CI	p-value
Intercept	249.91	13.55	223.34 – 276.48	<.001*
Time	-31.28	4.28	-39.66 – -22.89	<.001*
Day	-7.32	4.39	-15.92 – 1.28	.097
Condition	-8.13	6.06	-20.01 – 3.75	.200
Time by Day	12.64	6.15	0.58 – 24.70	.041*
Time by Condition	0.74	5.96	-10.94 – 12.42	.902
Day by Condition	16.13	6.04	4.29 – 27.96	.008*
Time by Day by Condition	-11.83	8.53	-28.55 – 4.90	.167
	**Voluntary activation [VA] (%)**			
Parameter	β	SE	95% CI	p-value
Intercept	92.48	1.90	88.76 – 96.20	<.001*
Time	-2.26	1.32	-4.85 – 0.34	.089
Day	1.79	1.36	-0.87 – 4.45	.188
Condition	0.87	1.31	-1.70 – 3.45	.508
Time by Day	-1.64	1.90	-5.37 – 2.09	.389
Time by Condition	-2.79	1.85	-6.42 – 0.84	.133
Day by Condition	-2.10	1.86	-5.75 – 1.55	.261
Time by Day by Condition	4.30	2.64	-0.86 – 9.47	.104
	**Potentiated twitch [Pt] (N·m)**			
Parameter	β	SE	95% CI	p-value
Intercept	68.83	3.77	61.44 – 76.22	<.001*
Time	-20.04	1.78	-23.52 – -16.56	<.001*
Day	-1.63	1.82	-5.20 – 1.94	.371
Condition	-0.36	1.76	-3.82 – 3.09	.838
Time by Day	2.01	2.54	-2.98 – 6.99	.431
Time by Condition	5.66	2.47	0.81 – 10.50	.023*
Day by Condition	1.78	2.50	-3.11 – 6.67	.477
Time by Day by Condition	-1.14	3.53	-8.06 – 5.77	.746
	**Contraction duration [CD] (ms)**			
Parameter	β	SE	95% CI	p-value
Intercept	326.84	0.11	260.95 – 409.38	<.001*
Time	0.77	0.03	0.72 – 0.82	<.001*
Day	1.04	0.03	0.97 – 1.10	.269
Condition	0.88	0.08	0.75 – 1.03	.144
Time by Day	0.99	0.05	0.91 – 1.09	.882
Time by Condition	1.04	0.04	0.95 – 1.13	.437
Day by Condition	0.99	0.04	0.90 – 1.08	.781
Time by Day by Condition	1.00	0.06	0.88 – 1.14	.972
	**Rate of torque development [RTD] (N·m·s^{-1})**			
Parameter	β	SE	95% CI	p-value
Intercept	890.14	59.24	774.04 – 1006.24	<.001*
Time	-274.67	16.87	-307.73 – -241.62	<.001*
Day	-41.64	17.29	-75.54 – -7.74	.017*
Condition	-14.86	41.81	-96.81 – 67.10	.732
Time by Day	35.75	24.16	-11.61 – 83.10	.140
Time by Condition	82.36	23.48	36.33 – 128.38	.001*
Day by Condition	41.78	24.40	-6.03 – 89.60	.088
Time by Day by Condition	-33.77	34.24	-100.88 – 33.34	.325
	**Rate of relaxation [RR] (N·m·s^{-1})**			
Parameter	β	SE	95% CI	p-value
Intercept	471.24	0.14	359.03 – 618.51	<.001*
Time	0.85	0.06	0.76 – 0.95	.004*
Day	0.97	0.06	0.87 – 1.09	.614
Condition	1.06	0.14	0.81 – 1.39	.674
Time by Day	1.23	0.08	1.04 – 1.44	.013*
Time by Condition	1.37	0.08	1.17 – 1.60	<.001*

Day by Condition	1.04	0.08	0.89 – 1.22	.590
Time by Day by Condition	0.80	0.11	0.64 – 1.00	.048*
	**Half relaxation time [0.5RT] (ms)**			
Parameter	β	SE	95% CI	p-value
Intercept	67.22	0.10	55.02 – 82.14	<.001*
Time	0.84	0.04	0.78 – 0.91	<.001*
Day	1.07	0.04	0.98 – 1.16	.122
Condition	1.01	0.10	0.83 – 1.22	.941
Time by Day	0.82	0.06	0.73 – 0.92	.001*
Time by Condition	0.80	0.06	0.72 – 0.90	<.001*
Day by Condition	0.93	0.06	0.83 – 1.04	.199
Time by Day by Condition	1.27	0.08	1.08 – 1.49	.004*
	**V-M_{max}**			
Parameter	β	SE	95% CI	p-value
Intercept	0.24	0.14	0.18 – 0.32	<.001*
Time	0.96	0.13	0.74 – 1.24	.744
Day	1.00	0.14	0.75 – 1.32	.995
Condition	0.87	0.19	0.60 – 1.27	.486
Time by Day	0.85	0.20	0.58 – 1.26	.418
Time by Condition	0.99	0.19	0.69 – 1.42	.948
Day by Condition	1.32	0.19	0.90 – 1.93	.155
Time by Day by Condition	0.94	0.27	0.55 – 1.61	.831
	**EMG-M_{max}**			
Parameter	β	SE	95% CI	p-value
Intercept	0.07	0.01	0.05 – 0.08	<.001*
Time	-0.00	0.00	-0.01 – 0.01	.900
Day	-0.01	0.00	-0.01 – 0.00	.092
Condition	-0.01	0.01	-0.03 – -0.00	.036*
Time by Day	-0.00	0.00	-0.01 – 0.01	.452
Time by Condition	-0.00	0.00	-0.01 – 0.01	.654
Day by Condition	0.01	0.00	-0.00 – 0.02	.059
Time by Day by Condition	0.00	0.01	-0.01 – 0.02	.449
	**V-wave (mV)**			
Parameter	β	SE	95% CI	p-value
Intercept	1.63	0.21	1.22 – 2.03	<.001*
Time	-0.34	0.19	-0.70 – 0.03	.069
Day	0.63	0.20	0.23 – 1.02	.002*
Condition	0.35	0.55	-0.72 – 1.43	.539
Time by Day	-0.52	0.28	-1.06 – 0.02	.062
Time by Condition	-0.03	0.26	-0.54 – 0.48	.912
Day by Condition	-0.54	0.27	-1.07 – -0.01	.047*
Time by Day by Condition	0.34	0.38	-0.41 – 1.10	.373
	**M-wave (mV)**			
Parameter	β	SE	95% CI	p-value
Intercept	6.32	0.89	4.58 – 8.06	<.001*
Time	-1.08	0.59	-2.24 – 0.07	.067
Day	1.66	0.60	0.48 – 2.84	.006*
Condition	2.43	0.58	1.29 – 3.58	<.001*
Time by Day	-0.73	0.85	-2.39 – 0.94	.394
Time by Condition	-1.18	0.82	-2.79 – 0.43	.153
Day by Condition	-2.14	0.83	-3.78 – -0.51	.011*
Time by Day by Condition	1.69	1.20	-0.65 – 4.04	.158

* Statistically important effect. SE = standard error; CI = confidence interval; EMG = electromyography.
Time = before or after 60min of cycling; Day = training day one or five; Condition = HA or CON.

Output 6. Physiological, perceptual and performance variables before or during training days.

	**Variable**			
	**HR (beats·min^{-1})**			
Parameter	β	SE	95% CI	p-value
Intercept	137.56	3.99	129.73 – 145.39	<.001*
Cycletime^1	266.52	10.89	245.18 – 287.86	<.001*
Cycletime2	-83.80	10.88	-105.12 – -62.49	<.001*
Cycletime^3	43.45	10.90	22.09 – 64.82	<.001*
Day	-2.94	0.37	-3.67 – -2.21	<.001*
Condition	14.83	5.65	3.77 – 25.90	.020*
Power (scaled)	6.29	0.25	5.79 – 6.78	<.001*
Cycletime^1 by Day	-57.43	15.53	-87.87 – -27.00	<.001*
Cycletime^2 by Day	5.69	15.46	-24.62 – 36.00	.713
Cycletime^3 by Day	-6.81	15.41	-37.02 – 23.40	.659
Cycletime^1 by Condition	270.30	14.99	240.93 – 299.67	<.001*
Cycletime^2 by Condition	-45.27	14.96	-74.59 – -15.95	.003*
Cycletime^3 by Condition	-8.80	14.95	-38.10 – 20.50	.556
Day by Condition	-5.25	0.51	-6.26 – -4.24	<.001*
Cycletime^1 by Day by Condition	-61.34	21.46	-103.39 – -19.28	.004*
Cycletime^2 by Day by Condition	17.95	21.42	-24.04 – 59.95	.402
Cycletime^3 by Day by Condition	5.96	21.42	-36.02 – 47.94	.781
	**T_{re} (°C)**			
Parameter	β	SE	95% CI	p-value
Intercept	37.85	0.05	37.74 – 37.95	<.001*
Cycletime^1	15.21	0.34	14.54 – 15.88	<.001*
Cycletime^2	-2.95	0.34	-3.61 – -2.29	<.001*
Day	0.06	0.01	0.04 – 0.08	<.001*
Condition	0.23	0.06	0.11 – 0.36	.008*
Power (scaled)	-0.01	0.01	-0.03 – 0.01	.221
Cycletime^1 by Day	-0.87	0.49	-1.84 – 0.10	.078
Cycletime^2 by Day	0.30	0.49	-0.66 – 1.25	.545
Cycletime^1 by Condition	5.00	0.49	4.04 – 5.96	<.001*
Cycletime^2 by Condition	1.29	0.48	0.35 – 2.24	.007*
Day by Condition	-0.20	0.02	-0.24 – -0.17	<.001*
Cycletime^1 by Day by Condition	-0.81	0.71	-2.19 – 0.57	.252
Cycletime^2 by Day by Condition	-0.71	0.69	-2.07 – 0.65	.305
	**T_{sk} (°C)**			
Parameter	β	SE	95% CI	p-value
Intercept	32.03	0.25	31.53 – 32.52	<.001*
Cycletime^1	14.70	3.68	7.49 – 21.92	.001*
Cycletime^2	-14.49	0.70	-15.87 – -13.11	<.001*
Cycletime^3	3.08	0.68	1.75 – 4.42	<.001*
Day	0.33	0.02	0.28 – 0.38	<.001*
Condition	3.82	0.33	3.17 – 4.46	<.001*
Power (scaled)	0.05	0.02	0.02 – 0.08	<.001*
Cycletime^1 by Day	2.39	1.07	0.28 – 4.49	.026*
Cycletime^2 by Day	2.11	0.96	0.24 – 3.99	.027*
Cycletime^3 by Day	1.18	0.93	-0.65 – 3.01	.206
Cycletime^1 by Condition	6.50	5.17	-3.64 – 16.64	.230
Cycletime^2 by Condition	0.86	0.93	-0.96 – 2.69	.352
Cycletime^3 by Condition	4.12	0.91	2.34 – 5.90	<.001*
Day by Condition	-0.41	0.03	-0.47 – -0.35	<.001*
Cycletime^1 by Day by Condition	-4.35	1.39	-7.08 – -1.62	.002*
Cycletime^2 by Day by Condition	-2.74	1.28	-5.24 – -0.25	.032*
Cycletime^3 by Day by Condition	-0.13	1.26	-2.59 – 2.33	.919
	**RPE (AU) [analysed using beta regression]**			
Parameter	β	SE	95% CI	p-value
Intercept	0.73	0.12	0.57 – 0.92	.008*
Condition	0.78	0.17	0.55 – 1.09	.140
Day	1.00	0.17	0.71 – 1.39	.981

Cycletime	1.01	0.00	1.00 – 1.01	.005*
Condition by Day	1.28	0.24	0.79 – 2.06	.313
Condition by Cycletime	1.01	0.00	1.01 – 1.02	.002*
Day by Cycletime	1.00	0.00	0.99 – 1.01	.899
Condition by Day by Cycletime	0.99	0.01	0.98 – 1.00	.242
	**Power (Watts)**			
Parameter	β	SE	95% CI	p-value
Intercept	172.97	9.73	153.91 – 192.04	<.001*
Cycletime	-0.06	0.04	-0.14 – 0.02	.155
Day	3.37	2.03	-0.60 – 7.34	.096
Condition	4.74	3.30	-1.73 – 11.22	.171
Cycletime by Day	-0.00	0.06	-0.11 – 0.11	.978
Cycletime by Condition	-0.13	0.06	-0.25 – -0.02	.019*
Day by Condition	-5.22	2.86	-10.83 – 0.39	.068
Cycletime by Day by Condition	0.03	0.08	-0.13 – 0.19	.689
	**Cadence (revolutions·min^{-1})**			
Parameter	β	SE	95% CI	p-value
Intercept	79.83	2.71	74.52 – 85.14	<.001*
Cycletime	0.02	0.01	0.00 – 0.05	.044*
Day	-1.60	0.58	-2.73 – -0.47	.006*
Condition	-2.76	2.14	-6.95 – 1.44	.234
Cycletime by Day	-0.03	0.02	-0.06 – 0.01	.122
Cycletime by Condition	0.03	0.02	-0.00 – 0.06	.064
Day by Condition	2.45	0.82	0.84 – 4.05	.003*
Cycletime by Day by Condition	-0.06	0.02	-0.11 – -0.02	.007*
	**Baseline urine osmolality (mOsm·kg^{-11})**			
Parameter	β	SE	95% CI	p-value
Intercept	532	110	319 – 746	<.001*
Day	242	113	29 – 459	.047*
Condition	41	108	-164 – 246	.711
Day by Condition	-263	169	-584 – 56	.137
	**Baseline urine specific gravity (AU)**			
Parameter	β	SE	95% CI	p-value
Intercept	1.013	0.003	1.007 – 1.019	<.001*
Day	0.010	0.004	0.002 – 0.017	.024*
Condition	0.003	0.004	-0.004 – 0.011	.450
Day by Condition	-0.010	0.006	-0.021 – 0.001	.105
	**Sweat rate (L·h^{-1})**			
Parameter	β	SE	95% CI	p-value
Intercept	0.84	0.09	0.65 – 1.027	<.001*
Day	0.06	0.04	-0.02 – 0.13	.161
Condition	0.46	0.05	0.37 - 0.55	<.001*
Day by Condition	-0.06	0.05	-0.16 – 0.04	.291

* Statistically important effect.

SE = standard error; CI = confidence interval; HR = heart rate; T_{re} = rectal temperature; T_{sk} = skin temperature; RPE = rating of perceived exertion.

Cycletime = time during 60 min cycle; Day = training day one or five; Condition = HA or CON; Power = cycling power in Watts.
