# Supplementary figures and images for: Short-term heat acclimation preserves knee extensor torque but does not improve 20 km self-paced cycling performance in the heat
Source: Eur J Appl Physiol. 2021 Jun 19;121(10):2761–72. doi: 10.1007/s00421-021-04744-y (PMC8416835; doi:10.1007/s00421-021-04744-y)

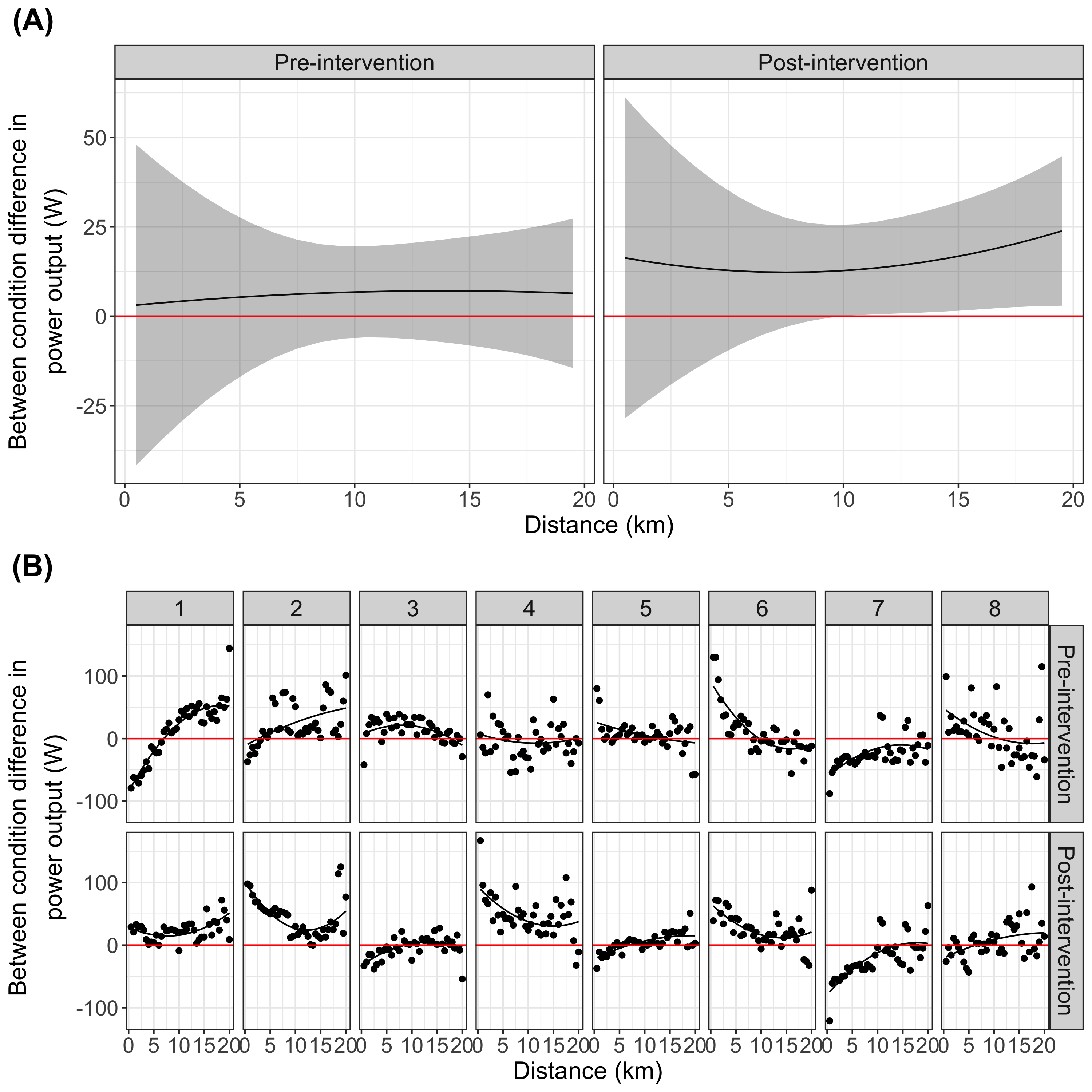

Supplement: Supplementary file 3 — Supplementary file3 (TIFF 35160 kb) [file 421_2021_4744_MOESM3_ESM.tiff]

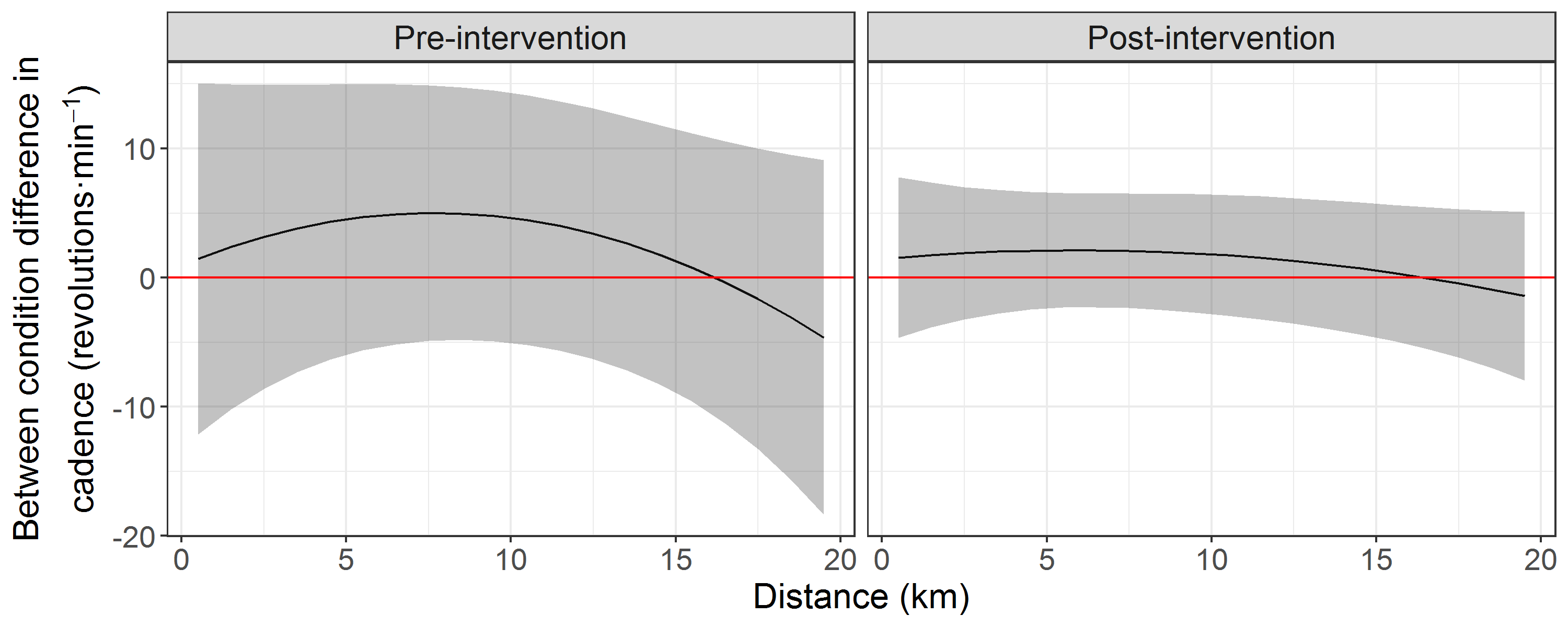

Supplement: Supplementary file 4 — Supplementary file4 (TIFF 114 kb) [file 421_2021_4744_MOESM4_ESM.tiff]

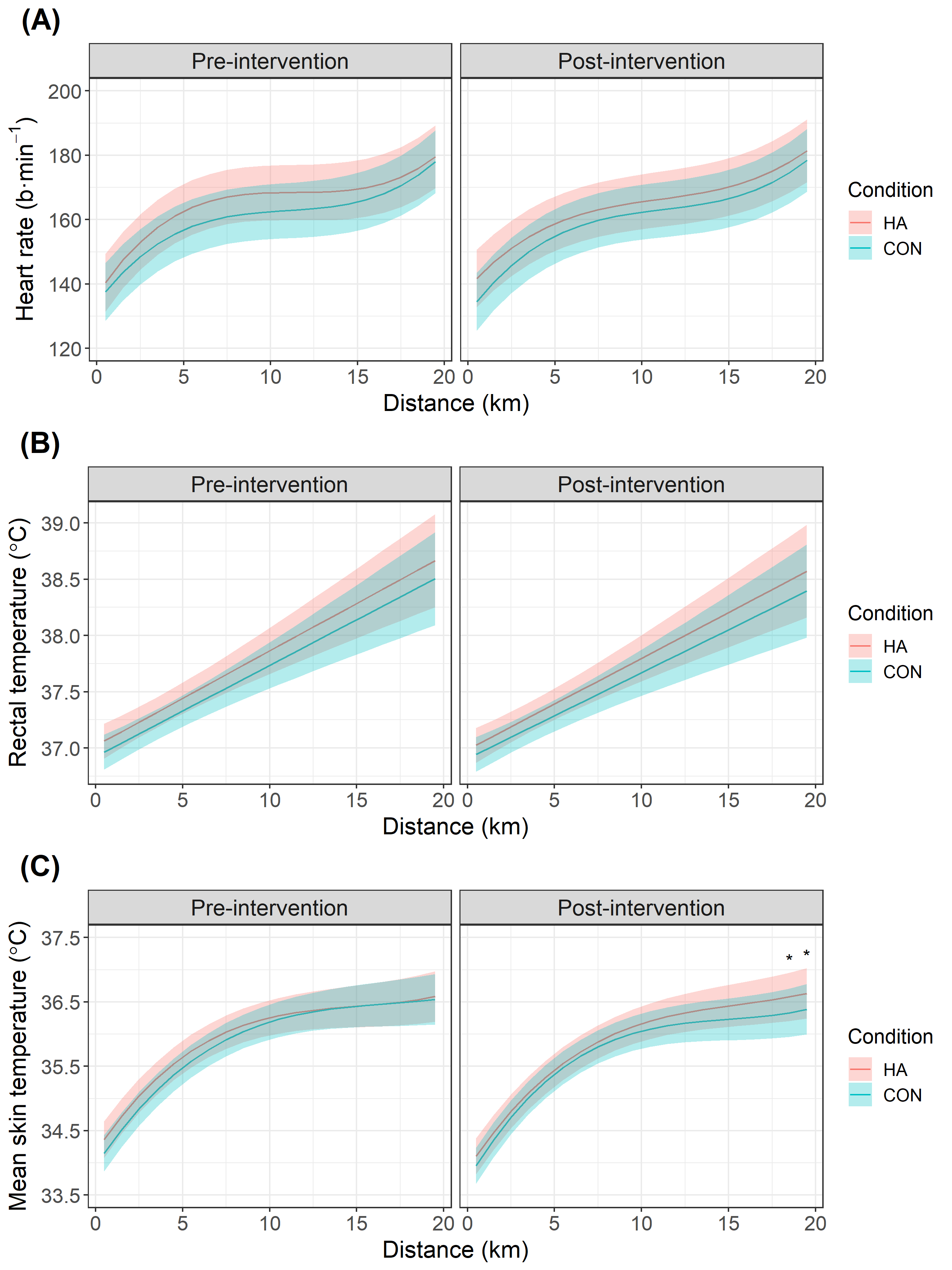

Supplement: Supplementary file 5 — Supplementary file5 (TIFF 403 kb) [file 421_2021_4744_MOESM5_ESM.tiff]

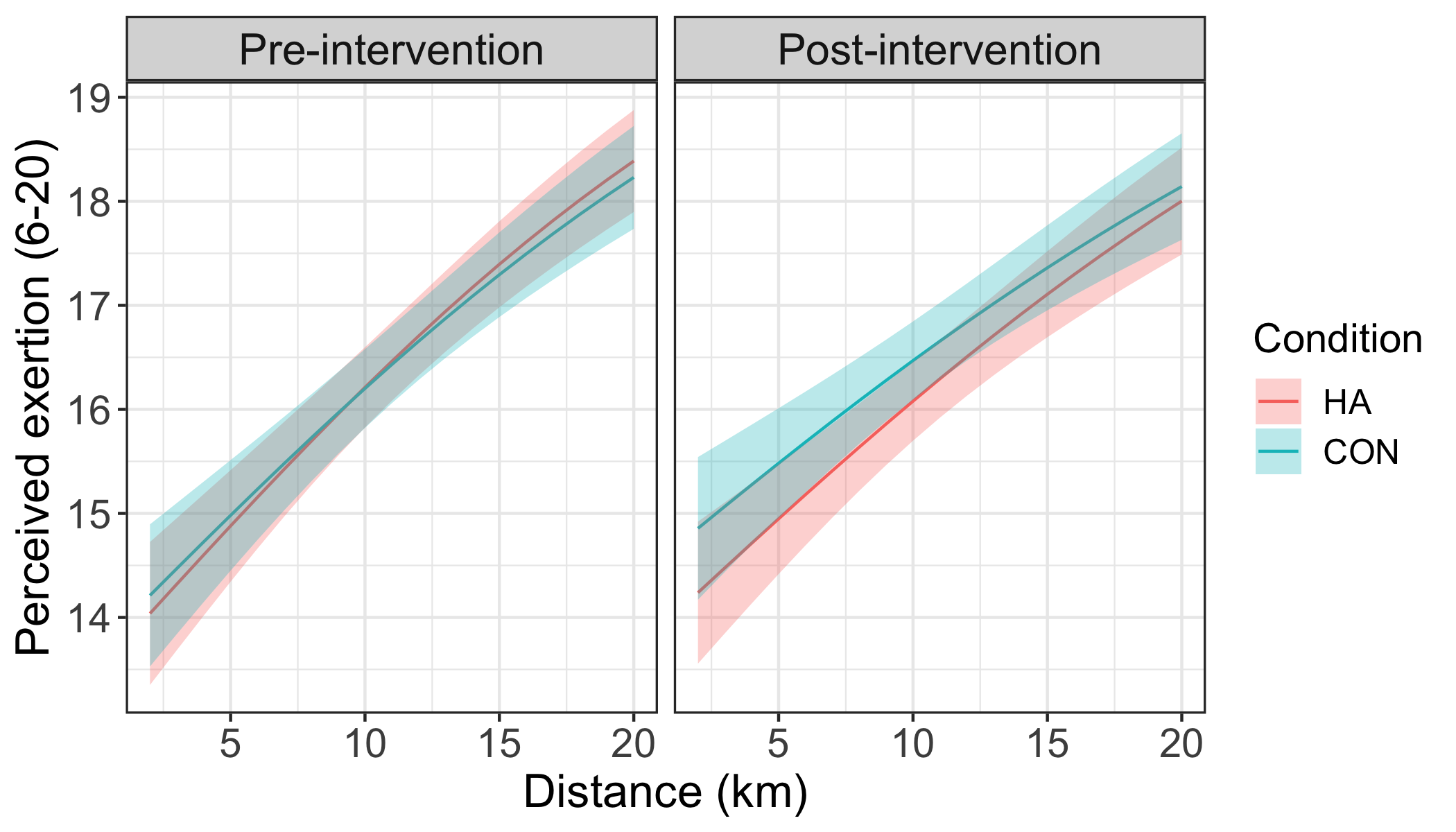

Supplement: Supplementary file 6 — Supplementary file6 (TIFF 9846 kb) [file 421_2021_4744_MOESM6_ESM.tiff]

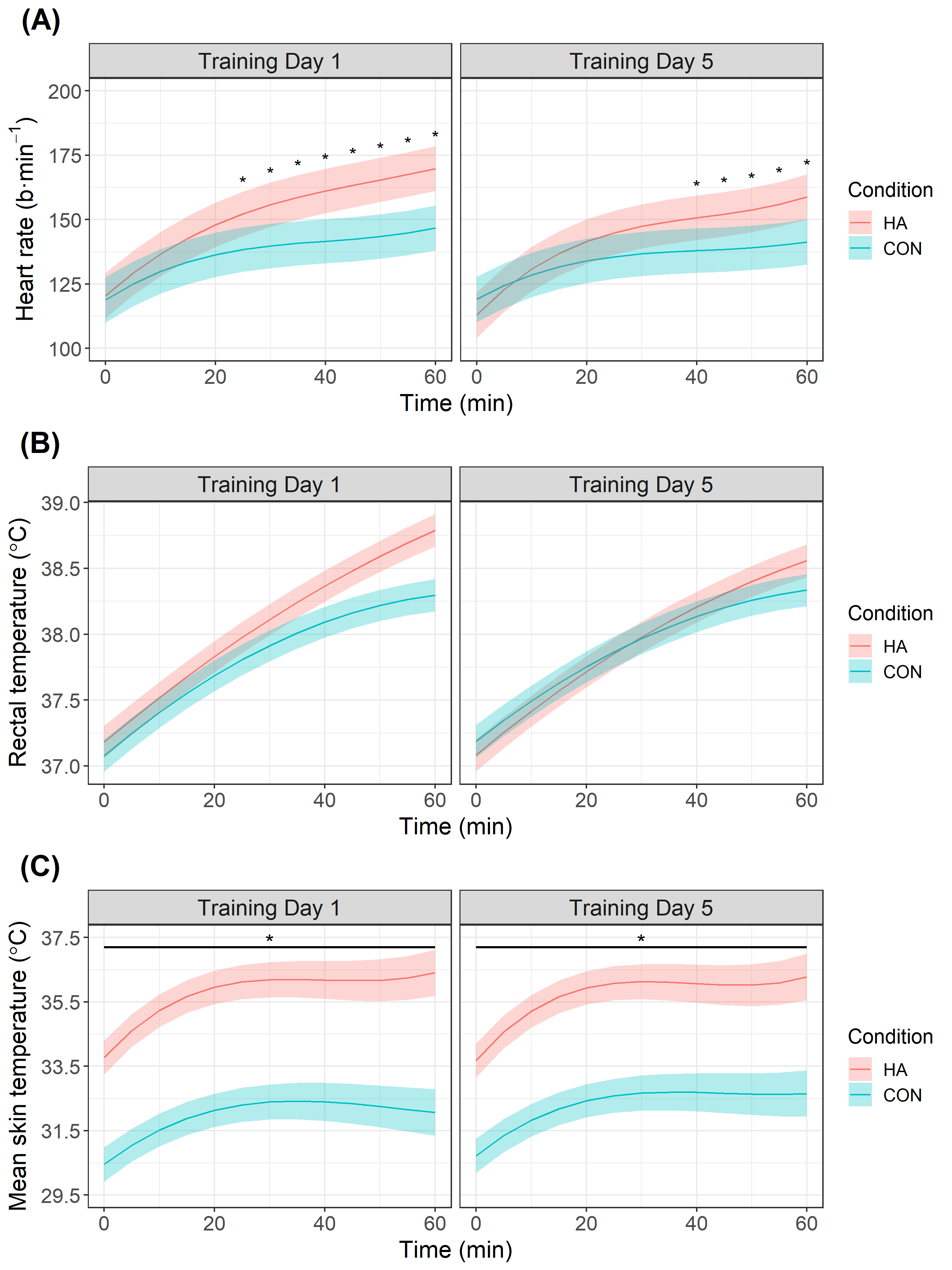

Supplement: Supplementary file 7 — Supplementary file7 (TIFF 358 kb) [file 421_2021_4744_MOESM7_ESM.tiff]

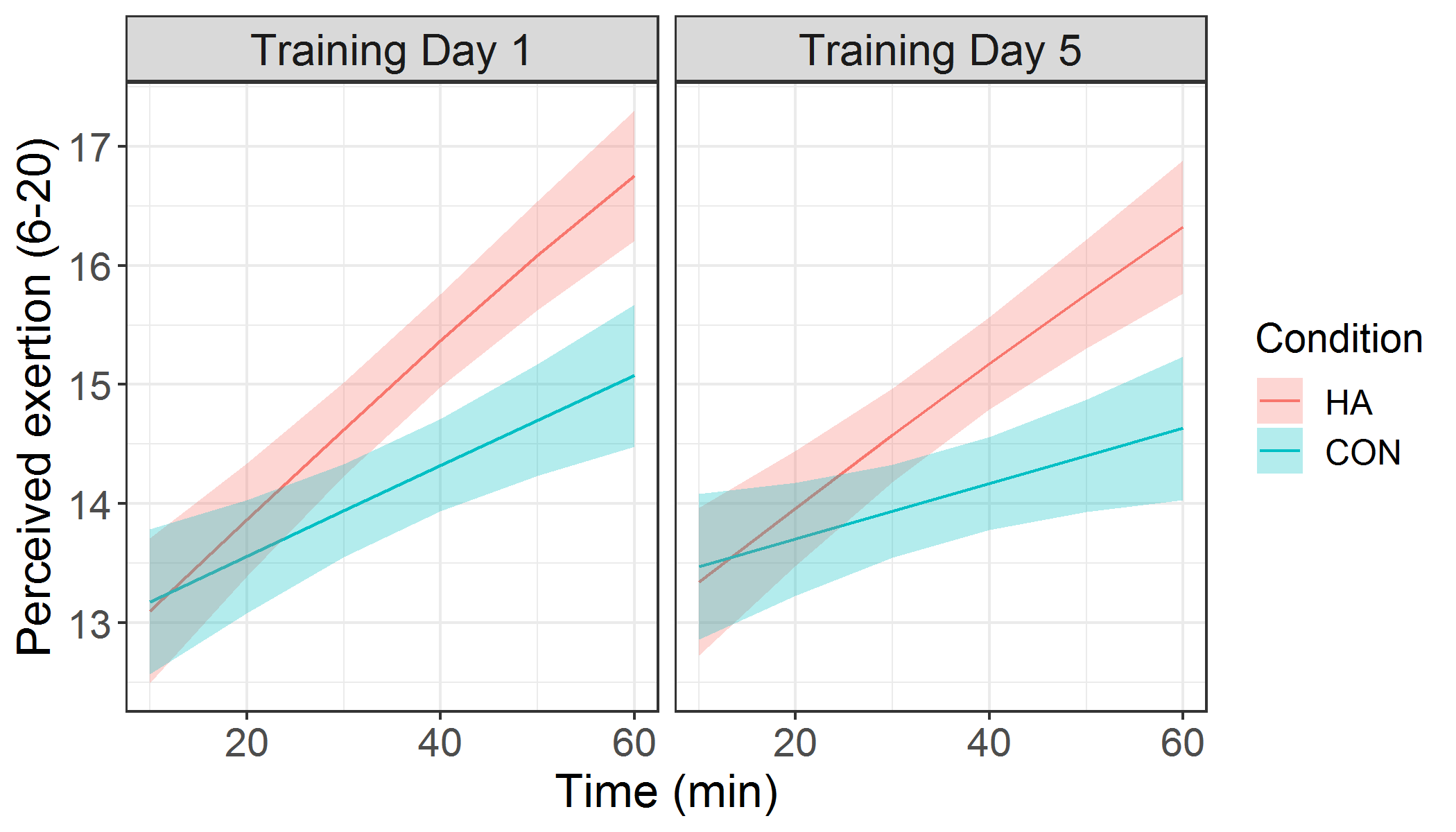

Supplement: Supplementary file 8 — Supplementary file8 (TIFF 113 kb) [file 421_2021_4744_MOESM8_ESM.tiff]
