# Supplementary material for: Short-term heat acclimation preserves knee extensor torque but does not improve 20 km self-paced cycling performance in the heat
Source: Eur J Appl Physiol. 2021 Jun 19;121(10):2761–72. doi: 10.1007/s00421-021-04744-y (PMC8416835; doi:10.1007/s00421-021-04744-y)
Supplement: Supplementary file 9 — Supplementary file9 (DOCX 24 kb) [file 421_2021_4744_MOESM9_ESM.docx]

Supplement 4 – Tables

**Table 1.** Knee extensor contractile variables before and after 20 km time trial performance.

| Variable | Condition | Pre-intervention | | Post-intervention | |
| --- | --- | --- | --- | --- | --- |
|  |  | Pre-cycling | Post-cycling | Pre-cycling | Post-cycling |
| M-wave (mV) | CON | 7.7 [6.0, 9.3] | 5.7 [4.0, 7.4] | 5.6 [4.0, 9.2] | 5.3 [3.6, 7.0] |
|  | HA | 8.5 [5.8, 11.1] | 6.0 [3.3, 8.6] | 6.5 [3.9, 9.2] | 5.4 [2.8, 8.1] |
| V-wave (mV) | CON | 1.72 [1.14, 2.60] | 1.17 [0.77, 1.78] | 1.32 [0.87, 1.99] | 0.85 [0.56, 1.31] |
|  | HA | 2.43 [1.38, 4.26] | 1.0 [0.57, 1.75] | 1.64 [0.93, 2.88] | 1.11 [0.63, 1.95] |
| CD (ms) | CON | 150 [130, 170] | 117 [96, 137] | 150 [130, 171] | 115 [95, 135] |
|  | HA | 149 [135, 163] | 114 [100, 128] | 148 [134, 162] | 111 [97, 125] |
| RTD (N∙m∙s^-1^) | CON | 845 [689, 1035] | 678 [553, 830] | 823 [672, 1009] | 702 [573, 861] |
|  | HA | 813 [721, 917] | 727 [645, 819] | 857 [760, 965] | 720 [638, 812] |
| RR (N∙m∙s^-1^) | CON | 543 [372, 714] | 550 [379, 721] | 480 [309, 651] | 542 [370, 713] |
|  | HA | 533 [433, 633] | 550 [450, 650] | 470 [371, 570] | 517 [417, 617] |
| 0.5RT (ms) | CON | 70 [51, 88] | 48 [29, 67] | 73 [54, 92] | 48 [29, 66] |
|  | HA | 65 [55, 76] | 47 [37, 58] | 71 [61, 82] | 47 [36, 57] |

Note: values reported as mean [95% confidence interval]. CON = control; HA = heat acclimation; CD = contraction duration; RTD = rate of torque development; RR = rate of relaxation; 0.5RT = half relaxation time.

**Table 2.** Neuromuscular variables before and after training days 1 and 5.

| Variable | Condition | Day 1 |  | Day 5 |  |
| --- | --- | --- | --- | --- | --- |
|  |  | Pre-cycling | Post-cycling | Pre-cycling | Post-cycling |
| MVC (N∙m) | CON | 250 [211, 289] | 219 [179, 258] | 243 [203, 282] | 224 [185, 263] |
|  | HA | 242 [202, 282] | 211 [171, 252] | 251 [210, 291] | 221 [181, 261] |
| VA (%) | CON | 92.5 [87.7, 97.5] | 90.2 [85.2, 95.2] | 94.3 [89.2, 99.3] | 90.4 [85.4, 95.4] |
|  | HA | 93.4 [88.4, 98.4] | 88.3 [83.3, 93.3] | 93.0 [88.1, 98.0] | 90.7 [85.7, 95.7] |
| Pt (N∙m) | CON | 69 [58, 79] | 49 [38, 59] | 67 [57, 78] | 49 [39, 60] |
|  | HA | 69 [58, 79] | 54 [44, 65]^$^ | 69 [58, 79] | 55 [45, 66] ^$^ |
| M-wave (mV) | CON | 6.32 [3.95, 8.69] | 5.24 [2.86, 7.61] | 7.98 [5.60, 10.36] | 6.17 [3.79, 8.55] |
|  | HA | 8.75 [6.38, 11.12]* | 6.49 [4.12, 8.86]* | 8.27 [5.90, 10.64] | 6.97 [4.58, 9.36] |
| V-wave (mV) | CON | 1.63 [1.10, 2.15] | 1.29 [0.76, 1.82] | 2.25 [1.71, 2.80] | 1.40 [0.85, 1.94] |
|  | HA | 1.98 [0.59, 3.37] | 1.61 [0.22, 3.00] | 2.07 [0.68, 3.46] | 1.52 [0.13, 2.91] |
| V-M_max_ | CON | 0.242 [0.168, 0.348] | 0.232 [0.161, 0.333] | 0.242 [0.165, 0.354] | 0.197 [0.135, 0.287] |
|  | HA | 0.211 [0.123, 0.360] | 0.199 [0.117, 0.340] | 0.278 [0.163, 0.474] | 0.211 [0.123, 0.361] |
| EMG-M_max_ | CON | 0.062 [0.041, 0.094] | 0.059 [0.039, 0.090] | 0.057 [0.038, 0.086] | 0.053 [0.035, 0.080] |
|  | HA | 0.051 [0.039, 0.065] | 0.049 [0.038, 0.063] | 0.053 [0.041, 0.069] | 0.052 [0.040, 0.067] |
| CD (ms) | CON | 327 [234, 457] | 252 [180, 353] | 339 [242, 474] | 260 [186, 363] |
|  | HA | 287 [220, 374] | 229 [176, 298] | 293 [225, 383] | 233 [179, 304] |
| RTD (N∙m∙s^-1^) | CON | 890 [718, 1063] | 615 [443, 788] | 849 [676, 1021] | 610 [437, 782] |
|  | HA | 875 [780, 971] | 683 [587, 779] | 875 [780, 971] | 685 [589, 781] |
| RR (N∙m∙s^-1^) | CON | 471 [317, 700] | 400 [269, 594] | 458 [308, 680] | 477 [321, 709] |
|  | HA | 500 [439, 570] | 581 [510, 662]^#^ | 508 [445, 579] | 577 [506, 658] |
| 0.5RT (ms) | CON | 67 [50, 90] | 57 [42, 76] | 72 [54, 96] | 50 [37, 66] |
|  | HA | 67 [57, 81] | 46 [38, 55] | 67 [56, 81] | 47 [39, 57] |

Note: values reported as mean [95% confidence interval]. * = different to CON within the same day (averaged over levels of time). $ = different to CON within the same time (averaged over levels of day). # = different to CON within the same day, at the same time point.

CON = control; HA = heat acclimation; MVC = maximum voluntary contraction; VA = voluntary activation; Pt = potentiated twitch; EMG = electromyography; CD = contraction duration; RTD = rate of torque development; RR = rate of relaxation; 0.5RT = half relaxation time.
